# No evidence for the putative nitric oxide sensor NsrR as a key regulator of magnetosome formation in *Magnetospirillum gryphiswaldense*

**DOI:** 10.1093/nar/gkaf1422

**Published:** 2026-01-06

**Authors:** Alexandra Woller, René Uebe, Dirk Schüler

**Affiliations:** Department of Microbiology, University of Bayreuth, Bayreuth95447, Germany; Department of Microbiology, University of Bayreuth, Bayreuth95447, Germany; Department of Microbiology, University of Bayreuth, Bayreuth95447, Germany

## Abstract

Magnetotactic bacteria produce membrane-bound organelles known as magnetosomes, which consist of chains of magnetite crystals and function as sensors for orientation within the Earth’s magnetic field. Magnetosome biosynthesis is a complex, multistep process that depends on iron availability and suboxic conditions. However, the expression of the ca. 30 core magnetosome biosynthetic genes has previously been described as constitutive and largely unaffected by environmental conditions.

A recent study by Pang *et al.*, published in this journal, reported the identification of a transcriptional regulator, NsrR*_Mg_*, which was proposed to activate magnetosome biosynthesis in *Magnetospirillum gryphiswaldense* in response to the endogenous signaling molecule nitric oxide (NO). Furthermore, the study suggested that NO also regulates a putative, previously unrecognized nitrification pathway that presumably supports endogenous NO production via denitrification, even in the absence of extracellular nitrate.

Here, we present results of genetic and transcriptional analyses demonstrating that, contrary to the findings of Pang *et al.*, NsrR*_Mg_* is not required for magnetosome biosynthesis. We also refute the existence of the proposed nitrification pathway and conclude that there is no compelling evidence supporting a role of NsrR*_Mg_* as a key regulator of magnetosome formation in *M. gryphiswaldense*.

## Graphical Abstract

**Graphical Abstract:**
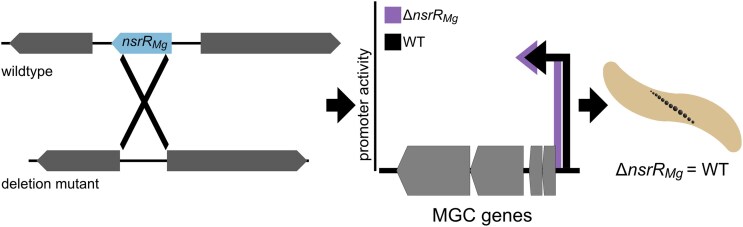

## Introduction

Magnetosomes are prokaryotic organelles that are formed by magnetotactic bacteria (MTB) as sensors for navigation in the Earth’s magnetic field [1–3]. In the well-studied alphaproteobacterium *Magnetospirillum gryphiswaldense (M. gryph.)* and related MTB, magnetosomes consist of tens-of-nanometer-sized crystals of the magnetic iron mineral magnetite (Fe_3_O_4_) that are aligned in well-ordered chains [4, 5]. Because of their exceptional characteristics, magnetosomes emerged as a model to study bacterial organelle biogenesis [6] and biomineralization [1, 7], but are also of interest in geosciences [8] and have potential for various biotechnological and biomedical applications [9–11]. Magnetosome biosynthesis proceeds by the invagination of magnetosome membrane (MM) vesicles from the cytoplasmic membrane, followed by sorting of specific magnetosome proteins into the MM, the transport of large amounts of iron into MM vesicles and biomineralization of monocrystalline Fe_3_O_4_ particles, and finally, their assembly and positioning into linear chains along a dedicated cytoskeletal network [3, 12, 13]. All biosynthesis steps are controlled mostly by >30 genes named *mam, mms* and *feoAB*, which are organized as magnetosome gene clusters (MGCs) and are necessary and sufficient to confer magnetosome biosynthesis to foreign bacteria [3, 14, 15].

Although microaerophilic magnetospirilla are able to tolerate higher oxygen concentrations for growth, magnetosome biosynthesis occurs only under anaerobic (i.e. denitrifying) to microaerobic conditions, whereas oxygen levels >2 kPa increasingly impair or even completely inhibit magnetite biomineralization [16–20]. However, transcriptomic studies comparing aerobically (nonmagnetic) and anaerobically grown (magnetic) *M. gryph*. cultures have shown that expression of biosynthetic MGCs is largely unaffected by oxygen levels [20–22]. Consistently, empty MM vesicles and magnetosome proteins encoded by the MGCs were present also in nonmagnetic cells under aerobic conditions [23,24]. Thus, while magnetosome genes are among the most highly transcribed genes under all conditions, they were shown to be essentially constitutively expressed.

The denitrification pathway (i.e. the stepwise reduction of NO_3_^−^ to N_2_ gas [25]) present in *M. gryphiswaldense* was shown to not only support anaerobic growth but also contribute to the maintenance of proper cellular redox balance (i.e. ferric-to-ferrous iron ratio) required for biomineralization of the mixed-valence iron oxide magnetite [18, 26, 27]. In contrast to MGCs, constituents of the denitrification pathway were found to be highly upregulated during anaerobic growth optimal for magnetite biomineralization [20, 21, 26]. However, although mutants of the periplasmic nitrate (Nap) or nitrite (NirS) reductases as well as the nitric oxide reductase (Nor) were impaired in magnetosome biosynthesis to variable degrees [15, 26, 27], they still formed magnetite, indicating that active denitrification is favorable, but not absolutely essential for biomineralization. Consistently, null mutants of the global anaerobic regulator FNR (fumarate and nitrate reduction), which derepresses nitrate respiration in the absence of oxygen [28–30], or of two oxidation-sensing OxyR-like regulators [31, 32], were somewhat impaired in magnetosome biosynthesis, but nevertheless continued to biomineralize magnetite. Likewise, starvation of cells for iron unsurprisingly reduces magnetite biomineralization, but does not affect expression of biosynthetic MGCs, and elimination of the conserved iron-responsive transcriptional regulators IrrB or Fur only weakly impaired magnetite formation [33, 34, 35]. Thus, the well-established phenotypic inhibition of magnetosome biosynthesis by high oxygen and low iron concentrations has been assumed to be largely independent of genetic regulation [20, 34].

Recently, a study by Pang *et al.* (2024) [36] reported the identification of the Rrf2-family transcriptional regulator NsrR*_Mg_*, a protein with homology to nitrous oxide (NO) sensors in other bacteria, as a possible key regulator of magnetosome formation in *M. gryph*. Strikingly, deletion of NsrR*_Mg_* was found to completely abolish magnetosome biosynthesis and to decrease transcription levels of several magnetosome genes. Indeed, EMSA (electrophoretic mobility shift assays) demonstrated binding of recombinant NsrR*_Mg_* to several promoters of magnetosome genes. NsrR*_Mg_* was also shown to repress the transcription of the well-known denitrification pathway, but to activate a presumable nitrification pathway suggested by Pang *et al.* based on bioinformatic prediction and isotope tracer experiments. Altogether, these findings led the authors to postulate a new hypothesis about the ancient function of magnetosomes that might have been in the detoxification of NO rather than as geomagnetic sensors, as well as on the evolution of NO signaling in biomineralization.

Homologs of NO-sensing NsrR regulators are present in a variety of both Gram-negative and -positive bacteria. Usually, in the absence of NO, NsrR acts as a repressor by binding to a specific DNA recognition site overlapping either transcription start sites (TSSs) or promoter sequences, thereby suppressing transcription of target genes [37]. When NO is present, it binds to a highly reactive [4Fe-4S]-cluster present in NsrR, which becomes converted to an inactive form that then releases from the DNA, enabling transcription [38–40]. In denitrifying bacteria such as *Neisseria meningitidis* and *Paracoccus denitrificans*, NsrR regulates genes encoding proteins for repair of damage caused by the toxic NO, and denitrification proteins that prevent NO buildup, including, for example, the NO reductase NorB, and the flavohemoglobin Hmp as the most conserved member of the NsrR regulon [37, 41–43]. In nondenitrifying bacteria such as *Escherichia coli* (*E. coli*), genes of the NsrR regulon are not only involved in NO detoxification, but also in general stress response, central carbon metabolism, molybdenum cofactor biosynthesis, motility, and biofilm formation [37, 38, 40, 44].

Owing to the inherent instability of [4Fe-4S]-clusters, NsrR is known to become inactivated not only by NO but also high O_2_ levels [45]. NsrR*_Mg_* would thus be anticipated to downregulate magnetosome gene expression under aerobic conditions, a scenario, which, however, is not supported by the available transcriptomic data indicating constitutive expression of MGCs as described above [20]. This apparent contradiction, together with the rather far-reaching hypotheses presented by Pang *et al.*, prompted us to reassess the function of *M. gryph*. NsrR in magnetosome biosynthesis. In contrast to the findings by Pang *et al.*, we demonstrate by genetic and transcriptional analysis that NsrR is not required for magnetosome biosynthesis. We also refute the existence of the presumable nitrification pathway suggested by Pang *et al.* and conclude that NsrR is NOT a key regulator of magnetosome formation in *Magnetospirillum gryphiswaldense*.

## Materials and methods

### Bacterial strains, plasmids, and growth conditions

Strains and plasmids used in this study are listed in Supplementary Table S1. *Magnetospirillum gryphiswaldense* MSR-1 (DSM No. 6361) and derivatives were routinely cultivated in nitrate-containing flask standard medium (here termed FSM-NO_3_^−^), containing 10 mM HEPES (pH 7.0), 15 mM potassium lactate, 4 mM sodium nitrate (NaNO_3_), 0.74 mM potassium phosphate (KH_2_PO_4_), 0.6 mM magnesium sulfate (MgSO_4_ × 7H_2_O), 50 µM iron citrate, 3 g/l soy peptone, 0.1 g/l yeast extract, in flasks containing 2% (vol/vol) O_2_ in the headspace, at 120 rpm agitation [18]. When indicated, NaNO_3_ was replaced by 4 mM NH_4_Cl (termed FSM-NH_4_^+^). For growth under anaerobic conditions, the concentration of NaNO_3_ (and for comparison, also NH_4_Cl) was increased to 8 mM. Selection for deletion mutants was carried out on solid FSM with 1.5% (wt/vol) agar and 5 mg/ml kanamycin (Km).

Modified sodium lactate medium (mSLM) containing (per liter) 2.25 g sodium lactate, 0.05 g sodium thioglycolate, 0.4 g NH_4_Cl, 0.5 g K_2_PO_4_, 0.1 g MgSO_4_ × 7H_2_O, and 5 ml of trace element mixture plus ferric citrate (added to mSLM at final concentration 60 μM) [31] was first used to precisely reconstruct growth conditions described by Pang *et al.* (2024) but was later exchanged by FSM as described above.

Growth experiments were carried out in Hungate tubes containing 10 ml FSM with either NaNO_3_ or NH_4_Cl as nitrogen source. When indicated, peptone was omitted from the medium to obtain peptone-free FSM_PF_. For aerobic conditions, cells were incubated in free gas exchange with air in 100 ml Erlenmeyer flasks containing 20 ml medium. For microaerobic growth, cells were incubated in Hungate tubes sealed with butyl rubber stoppers under a microoxic gas mixture containing 2% O_2_ and 98% N_2_ before autoclaving. Anaerobic conditions were achieved by omitting O_2_ from the gas mixture, and anaerobic growth was performed in anaerobic Hungate tubes (100% N_2_). Cells were inoculated at OD = 0.03 and incubated for 72 h at 22°C.

*Escherichia coli* DH5α and WM3064 strains carrying plasmids were cultivated in lysogeny broth (LB) supplemented with 25 mg/ml Km at 37°C, with 180 rpm agitation. For cultivation of WM3064, 0.1 mM DL-α, ε-diaminopimelic acid (DAP) was added in addition.

### Molecular and genetic techniques

Primers used in this study are listed in Supplementary Table S2. Oligonucleotides used as primers for amplification of DNA fragments were deduced from the genome sequence of *M. gryph*. (GenBank accession number CP027526 [46]) and purchased from Sigma–Aldrich (Steinheim, Germany). Plasmids were constructed by standard recombinant techniques as described below. Generated constructs were sequenced by Macrogen Europe (Amsterdam, Netherlands) and sequence data analyzed with Geneious Prime 2024 (https://www.geneious.com).

*Construction of markerless gene deletion mutants*. Generation of a *nsrR_Mg_* deletion mutant was accomplished by a tailored *galK* counterselection system as described previously [47]. The pOR-15a-*galK* vector was digested using EcoRV to insert fused fragments homologous to upstream and downstream regions of *nsrR_Mg_*, each of ~1 kb (amplified using primers AW394/396 and AW391/395, respectively). Proper construction of resulting plasmids was verified by PCR (RU841/1852) and sequencing. Suicide vectors harboring the deleted allele were transferred into *M. gryph*. strains by conjugation using *E. coli* WM3064 as donor. Genomic insertion mutants were identified using a kanamycin resistance marker [KmR, aminoglycoside 3′-phosphotransferase type IIa encoded by the aph(3′)-IIa gene] [48] which was present on the suicide vector. After 5 days of incubation at 28°C, KmR colonies were picked and re-grown in up to 1 ml FSM at 28°C. For generation of double crossover mutants, selected clones were plated on FSM agar containing 2.5 % galactose and 50 ng/ml anhydrotetracycline (Atet) to counterselect for vector integration by the lethal activity of galactokinase (GalK) induced by Atet. This enzyme catalyzes the phosphorylation of galactose [49, 50]. Since *M. gryph*. is unable to metabolize galactosephosphate, this product accumulates to toxic levels inside the cell. As a result, only cells that have undergone a second recombination event and thus, have lost the plasmid backbone, are able to survive [47]. Deletions were verified by polymerase chain reaction (PCR) (AW466/467 and AW468/469) and whole genome sequencing performed by Novogene GmbH (Munich, Germany). Random reinsertion of the kanamycin resistance gene was excluded by a spotting test on selective and nonselective FSM-agar plates. For complementation experiments, *nsrR_Mg_* and its putative promoter region were amplified from the *M. gryph*. genome using primers AW471/472. Insert and backbone vector pBBR1MCS-2 were digested by EcoRI and HindIII and then ligated. Ligation mixture was transferred to *E. coli* DH5α and successful cloning was confirmed by PCR and plasmid sequencing. Confirmed plasmids were transferred to *E. coli* WM3064, the donor for conjugation in *M. gryph*. Transformants were selected on FSM agar plates supplemented with kanamycin.

### Phenotypic analyses

Growth of *M. gryph*. cultures was determined by optical density (OD) measurements at 565 nm. The coefficient of magnetically induced differential light scattering and quantitative monitoring of cellular magnetism (Cmag) was determined as previously reported [51]. For visual evaluation of magnetite biomineralization and formation of larger colonies, strains were plated on thick agar plates with 140 ml of improved colony formation medium (ICFM), i.e. FSM supplied with an increased amount of iron (500 µM), in large-size (150 mm) Petri dishes at low seeding density (1 to 2 colonies per cm^2^) with an increased incubation time (14 days) at 28°C under microoxic conditions [15].

For the semi-quantitative denitrification assay, cells were inoculated to a final OD565 of ~0.02, mixed with FSM-NO_3_^−^ or FSM- NH_4_^+^ with 0.3% agar in oxygen gradient tubes and exposed to air [27].

### Nitrite analysis

For nitrite analysis, *M. gryph*. cells were grown under aerobic and anaerobic conditions at 27°C for 40–50 h. Nitrite was detected by using the modified Griess reagent (Sigma, Munich, Germany). Samples of 100 µl of cultures diluted 50-fold were reacted with an equal amount of modified Griess reagent, and after 15 min the absorbance at 540 nm was recorded. *M. gryph*. Δ*nirS* cultures were further diluted 500-fold prior to measurement. A nitrite standard curve (0–70 µM) was generated to calculate the final nitrite concentration.

### Transmission electron microscopy

For transmission electron microscopy (TEM) of cells, samples were fixed in 0.007% (wt/vol) formaldehyde and adsorbed onto carbon-coated copper-mesh grids (Science Services, Munich, Germany). TEM was performed on a JEOL 1400 (Japan) with an acceleration voltage of 80 kV and micrographs were analyzed using the software *ImageJ* [52].

### Luminescence measurements

Per strain and promoter, three randomly selected transconjugants harboring vector pBamII-Tn7-P-*luxAE* were analyzed in three technical replicates for luminescence. The luminescence signal was detected every 20 min as arbitrary light units by a multiwell plate reader equipped with a luminometer module (Infinite M200 PRO, Tecan, Männedorf, Switzerland) during growth of the cultures in FSM at 28°C and 280 rpm, over 200 cycles (72 h). Arbitrary light units were normalized to optical density measured at the wavelength of 565 nm (OD565) to obtain relative light units, according to the formula:


\begin{eqnarray*}
{\rm RLU} = {\mathrm{\ }}\frac{{{\rm Light}{\mathrm{\ }}{\rm AU}}}{{{\rm OD}565{\mathrm{\ }}{\rm AU}}}.
\end{eqnarray*}

Maxima of the RLU curves (RLUmax) were used to compare promoter activities.

### Bioinformatic analyses

For the detection of protein homologies, BLAST, Hidden Markov Model-based HHPred analyses [53] and CLuster Analysis of Sequences (CLANS) [54] were performed as described previously [55]. For phylogenetic analysis of the Rrf2 family, initial BLAST analyses with the characterized family members HypR from *Staphylococcus aureus* (WP_434175120.1), IscR from *E. coli* (NP_417026.1), CymR from *Bacillus subtilis* (NP_390630.2), RirS from *Sinorhizobium meliloti* (AEH77659.1), SaiR from *Bacillus anthracis* (WP_000093217.1), RsrR from Streptomyces *venezuelae* (WP_015037744.1), NsrR from *E. coli* (CUU96603.1), and SifR from *Streptococcus pneumoniae* (AVN86528.1) were performed to search for Rrf2 family homologs in the *M. gryph*. genome. Subsequently, a phylogenetic analysis was performed by aligning 147 protein sequences retrieved from the BLAST analyses using MAFFT 7.526 [56]. The alignment was trimmed (TrimAI 1.3, no gaps) [57] and then used to infer a maximum-likelihood tree with IQ-Tree 1.6.11 [58] under the LG + I + G4 model as suggested by ModelFinder [59]. Bootstrap support was derived by ultrafast bootstrap approximation with 1000 iterations. The phylogenetic tree was visualized and annotated using iTOL [60].

Genome comparison of two different MSR-1 genome versions (CP027526.1 and NC_023065.1, respectively) was done by dnadiff [61] and fastANI [62] analysis via the Galaxy server platform (version 25.0.3.dev0) [63]. For visualization of structural genome differences, a Mauve whole genome alignment was performed [64] and visualized using Circos [65].

### Statistical analyses

Statistical analyses were carried out as analyses of variance (ANOVA) and Tukey’s “Honest Significant Difference” (TukeyHSD) method using R version 4.5.1 [66]. Error bars in barplots show mean ± standard deviation (SD). Magnetosome numbers and sizes are given as mean ± SD.

## Results

### NsrR*_Mg_* is not required for magnetosome synthesis

Our analysis of the most recent *M. gryph*. genome sequence (CP027526 [46]) revealed the presence of seven genes encoding proteins of the Rrf2 family of transcriptional regulators (Supplementary Fig. S1). Notably, two of these proteins belong to the NsrR subfamily of NO-sensors: One of them, MSR1_14420, has 60.4% similarity to NsrR of *E. coli* and is 100% identical to MGMSRv2_0820 (genome NC_023065.1 [67]) referred to as NsrR*_Mg_* by Pang *et al.* (2024) (and in the following by us). A second gene (MSR1_19670), in the following referred to as *nsrR2*, encodes a protein with 58.6% sequence similarity to NsrR of *E. coli*, but only 54.1% to NsrR*_Mg_* (Supplementary Fig. S1). Homologues of *nsrR_Mg_* and *nsrR2* are also present in related *Magnetospirillum* species, but their genomic context is only partially conserved (not shown). To exclude the possibility that NsrR2 might interfere with NsrR*_Mg_* function, we constructed markerless *in frame* deletions of *nsrR2* in both WT and Δ*nsrR_Mg_* (see below) backgrounds, resulting in strains Δ*nsrR2* and Δ*nsrR_Mg_*Δ*nsrR2*. However, deletion of *nsrR2* had no obvious phenotype in both strains (Supplementary Fig. S2), therefore *nsrR2* was not considered further in this study.

**Figure 1.**
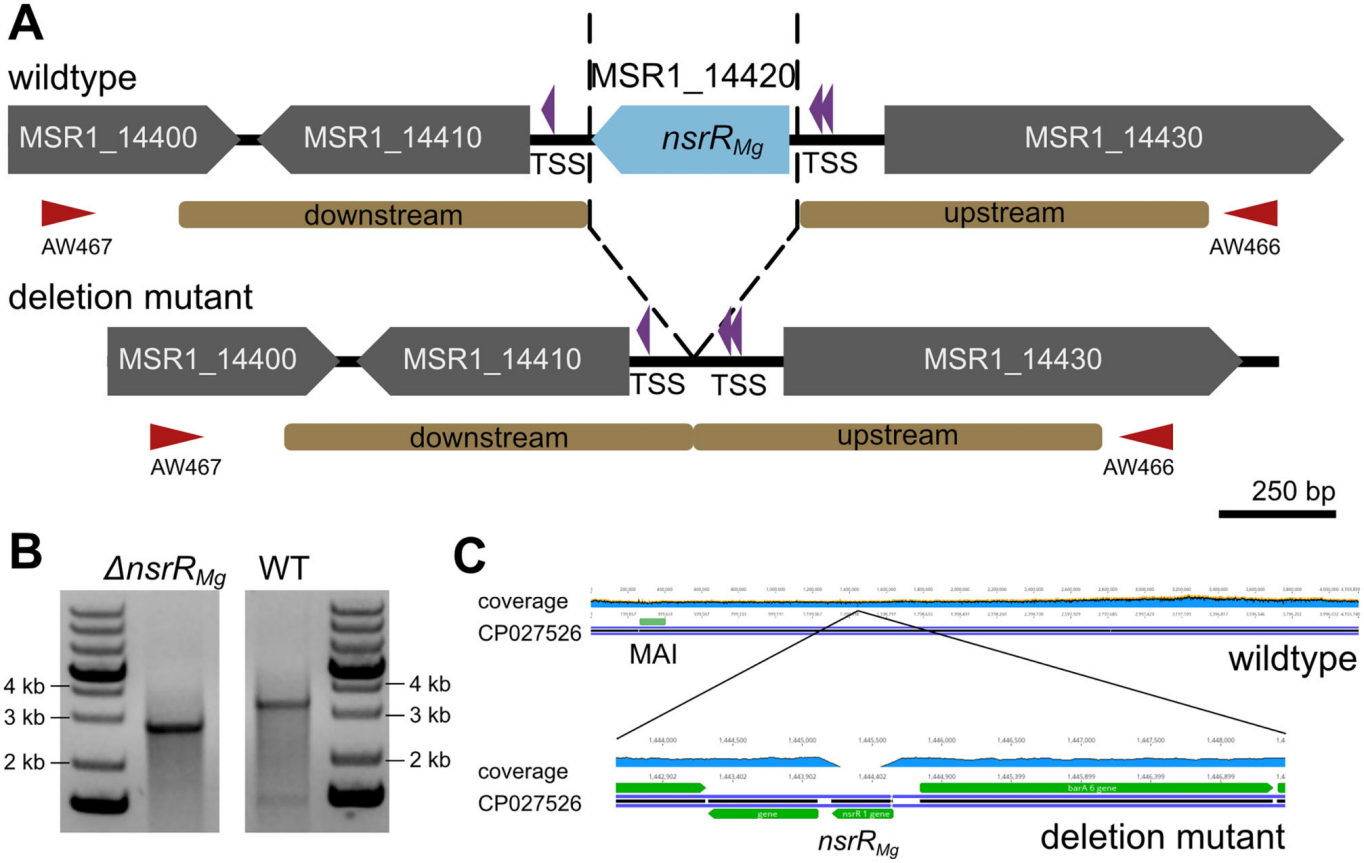
Genotype of ∆*nsrR_Mg_*. (**A**) Molecular organization of the genomic region of *nsrR_Mg_* (blue) before (WT, upper part) and after (lower part) its deletion. Downstream and upstream homologous regions on pOR-15a-*galK-nsrR_Mg_* used for allelic replacement by recombination are indicated in brown, and flanking primers are indicated as red arrow heads. Violet arrow heads indicate transcription start sites (TSSs, identified by Cappable-sequencing in a previous study [20]). (**B**) Agarose gel electropherogram showing the sizes of amplicons obtained by PCR screening with flanking primers (expected sizes for WT = 3,192 bp, *nsrR_Mg_* deletion = 2,754 bp).(**C**) Mapping of whole genome sequencing reads of Δ*nsrR_Mg_* (clone 1) to the *M. gryph*. WT sequence (CP027526.1). The locus of the magnetosome island (MAI) with all *M. gryph*. MGCs is indicated. The coverage is shown for the whole genome (top, 278.8 ± 62.1 mean coverage) and the *nsrR_Mg_* region (bottom, 19.7 ± 34.5 mean coverage). Lack of read coverage at the *nsrR_Mg_* locus (position 1,444,114 - 1,444,551 of the genome) confirms its deletion.

In *M. gryph*., *nsrR_Mg_* precedes a gene encoding a putative response regulator (MSR1_14410) with both genes transcribed in the same direction. However, both MSR1_14410 and *nsrR_Mg_* are likely transcribed independently from their own promoters, as TSSs were detected not only upstream of *nsrR_Mg_*, but also in front of MSR1_14410 within the ∼100 bp intergenic region between MSR1_14410 and *nsrR_Mg_* [20] (Fig. 1A). MSR1_14430, a putative signal transduction histidine-protein kinase gene, is encoded upstream of *nsrR_Mg_*, but transcribed in the opposite direction, altogether suggesting that *nsrR_Mg_* is transcribed monocistronically (Fig. 1A).

In the deletion mutant reported by Pang *et al.* (2024), the *nsrR_Mg_* gene was replaced by a gentamicin (Gm)-resistance cassette resulting in a strain hereafter named *nsrR_Mg_::GmR*. However, in this scenario putative polarity effects cannot be excluded. Therefore, we constructed a markerless *in-frame* mutant via homologous recombination [47, 68], without leaving foreign DNA or scars in the genome (Fig. 1A). After conjugation and counterselection, we obtained numerous *M. gryph*. clones, in which the expected genotype was confirmed by PCR (Fig. 1B). Genome resequencing of two selected clones verified the proper *nsrR_Mg_* deletion as well as the absence of second-site mutations within magnetosome genes (Fig. 1C). One of the clones, in the following named Δ*nsrR_Mg_*, was selected for further analysis.

We assessed growth and magnetosome formation of ∆*nsrR_Mg_* under various cultivation conditions and compared it to the wild type (WT) and the nonmagnetic mutant M05, which is entirely devoid of magnetosomes due to large deletions in the magnetosome island (MAI) including *mms6op, mamGFDCop, mamABop* and *mamXYop* (*op* for operons) [68]. In our hands, under both aerobic and microaerobic conditions, both WT and Δ*nsrR_Mg_* essentially failed to grow (i.e. only to an OD of 0.03 – 0.04 and a Cmag of 0.3 – 0.6 (Supplementary Tables S3 and S4)) in the modified sodium lactate medium (mSLM [31]) used by Pang *et al.* (2024), which contains NH_4_^+^ as the sole nitrogen source. Therefore, in further experiments, all *M. gryph*. strains were grown in FSM medium as used in ours and other labs for routine cultivation of *M. gryph*., with either NO_3_^−^ (= FSM-NO_3_^−^) or NH_4_^+^ (= FSM-NH_4_^+^) as sole nitrogen sources [18, 26, 69]. Under aerobic, microaerobic and anaerobic conditions, and with either NO_3_^−^ or NH_4_^+^, growth of ∆*nsrR_Mg_* was indistinguishable from that of WT, although all strains grew to lower final ODs in FSM-NH_4_^+^ than in FSM-NO_3_^−^ under microoxic conditions. As expected, neither WT nor ∆*nsrR_Mg_* grew anaerobically in FSM-NH_4_^+^ due to the absence of nitrate as respiratory electron acceptor for denitrification [26] (Supplementary Table S3).

**Figure 2.**
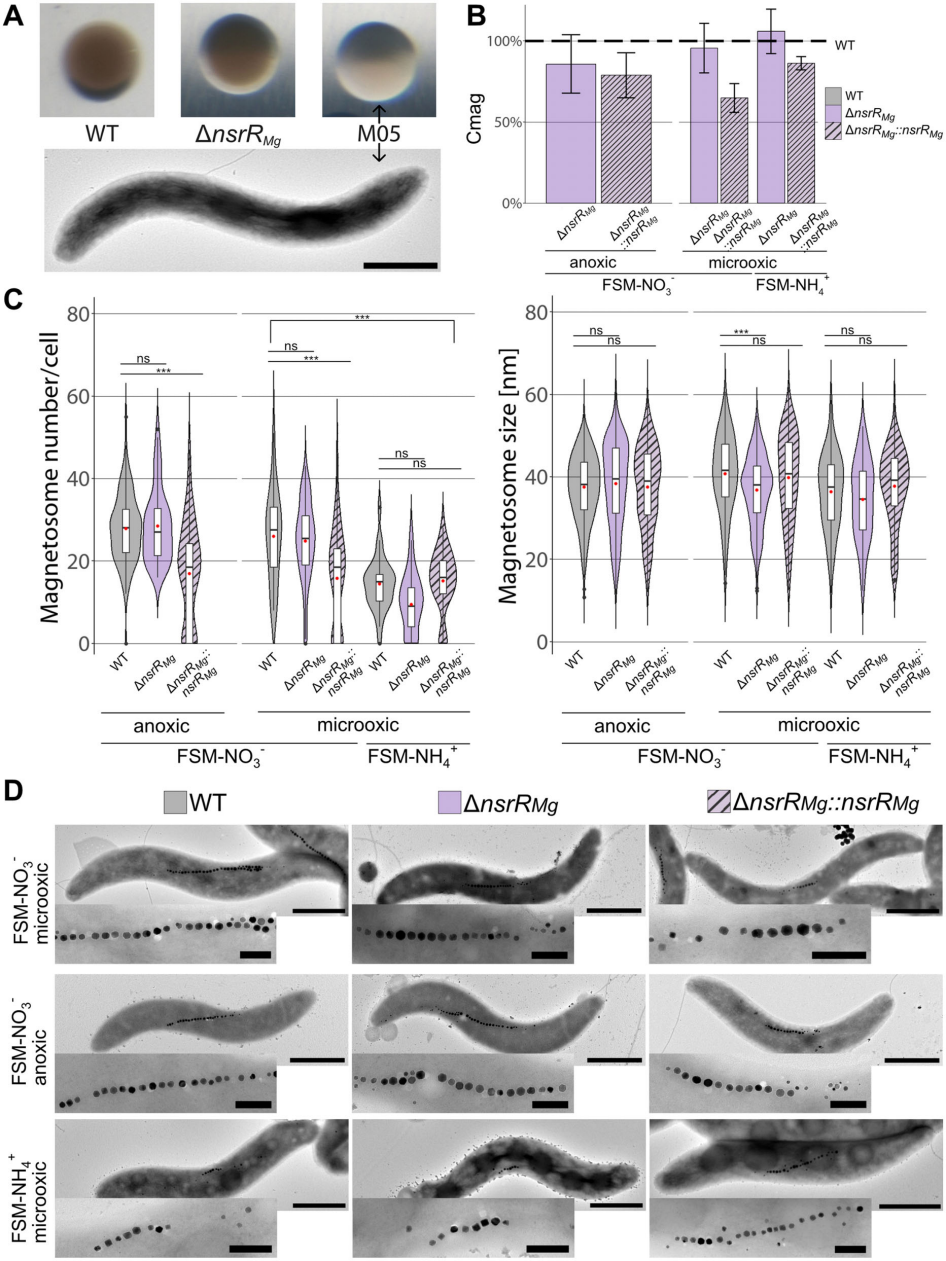
Phenotypic analysis of Δ*nsrR_Mg_* and Δ*nsrR_Mg_::nsrR* compared to WT and nonmagnetic mutant M05. (**A**) Colony appearance of WT (left), nonmagnetic control M05 (right) and Δ*nsrR_Mg_* (middle) on thick agar plates after incubation under microoxic conditions. TEM shows M05 cell. (**B**) Magnetic response of Δ*nsrR_Mg_* (purple) and Δ*nsrR_Mg_::nsrR* (stripes) given as percent of WT Cmag (100%) under different growth conditions. Data presented for three biological replicates. (**C**) Violin plots displaying magnetosome number per cell (left) and magnetosome size (right) of WT (gray), Δ*nsrR_Mg_* (purple) and Δ*nsrR_Mg_::nsrR* (stripes). Under microoxic conditions, crystal sizes ranged between 35 ± 9 nm, 36 ± 9 nm and 38 ± 9 nm in FSM-NH_4_^+^ and 37 ± 8, 41 ± 9 nm and 40 ± 11 nm in FSM-NO_3_^−^ for ∆*nsrR_Mg_*, WT and ∆*nsrR_Mg_::nsrR*, respectively. Under anoxic conditions, magnetosomes had sizes of 38 ± 10 nm in both, ∆*nsrR_Mg_* and ∆*nsrR_Mg_::nsrR*, and 37 ± 8 nm in WT. Significance values were calculated by TukeyHSD post-hoc test; ***, *P* < 0.001; ns, not significant. Boxplots within violin plots display the minimum, maximum, and median of each data set. Red points indicate mean. (**D**) TEM images of WT (left), Δ*nsrR_Mg_* (middle) and Δ*nsrR_Mg_::nsrR* (right) with close-up of magnetosome chain in microoxic FSM-NO_3_^−^ (top), anoxic FSM-NO_3_^−^ (middle) and microoxic FSM-NH_4_^+^ (bottom). Scale bars: 1 µm (whole cell), 200 nm (close-up).

On solid FSM-NO_3_^−^ medium, ∆*nsrR_Mg_* formed brown-colored colonies, slightly varying in intensity from intermediate to dark, and very similar to magnetite-loaded WT colonies, but strikingly distinct to nonmagnetic M05, forming cream-colored colonies owing to the absence of magnetite [15] (Fig. 2A). Consistently, ∆*nsrR_Mg_* grown in liquid FSM-NH_4_^+^ under microaerobic and in liquid FSM-NO_3_^−^ under micro- and anaerobic conditions clearly showed a magnetic response, i.e. cells aligned to a magnetic field under the microscope and showed WT-like Cmag values (FSM-NO_3_^−^/microoxic: 96% of WT, FSM-NO_3_^−^/anoxic: 85% of WT, and FSM-NH_4_^+^/microoxic: 106% of WT) (Fig. 2B). In FSM-NO_3_^−^, under microoxic and anoxic conditions, ∆*nsrR_Mg_* cells contained similar numbers of magnetosomes as the WT (25 ± 10 and 28 ± 9 versus 26 ± 11 and 28 ± 8, respectively), whereas ∆*nsrR_Mg_::nsrR* cells had fewer magnetosomes under these conditions (microoxic: 16 ± 12, anoxic: 17 ± 12). All strains also formed magnetosomes in FSM-NH_4_^+^, if also fewer compared to FSM-NO_3_^−^ (Fig. 2C and D, and Supplementary Table S5). Under all tested growth conditions, also the size of magnetosomes was very similar in mutants compared to the WT (Fig. 2C and Supplementary Table S5).

The FSM medium used by us also contains soy peptone, which provides a growth-promoting effect [18], but is absent from the mSLM medium [31] exclusively used by Pang *et al.* (2024), who failed to detect any magnetosomes in *nsrR_Mg_::GmR* cells under undefined microoxic conditions. To exclude the possibility that magnetosome formation in ∆*nsrR_Mg_* somehow depends on the presence of peptone, we also tested a minimal, peptone-free (PF) medium based on FSM (FSM_PF_-NO_3_^−^ and FSM_PF_-NH_4_^+^). Growth of all strains in FSM_PF_-NO_3_^−^ was similar to that in FSM-NO_3_^−^, but severely impaired in FSM_PF_-NH_4_^+^ (Supplementary Table S3). Although Cmag values were reduced compared to FSM (Supplementary Table S4), all strains still biosynthesized magnetosomes (WT-like magnetosome numbers in FSM-NO_3_^−^ (Supplementary Fig. S3)), but with an increased portion of magnetosome-free cells in FSM_PF_-NH_4_^+^.

### NsrR*_Mg_* does not activate transcription of MGC genes

**Figure 3.**
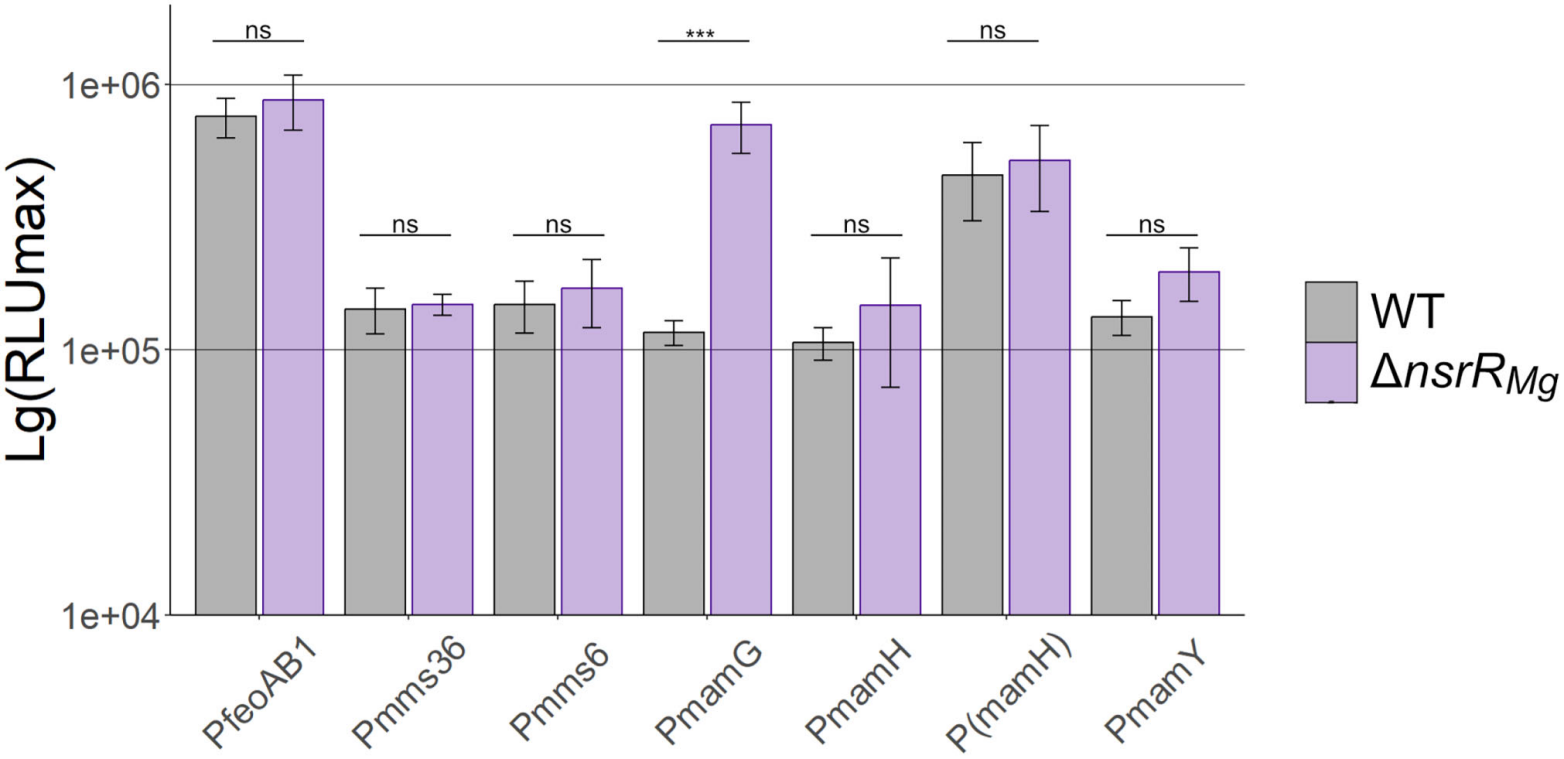
Results of bioluminescence assays of magnetosome gene promoters in *M. gryph*. WT (gray) and Δ*nsrR_Mg_* (purple) background analyzed by *lux* reporter gene fusions. Activity is shown as maximum relative light units (RLU). Bars represent the mean of three biological replicates with error bars indicating the standard deviation. Significance values were calculated by TukeyHSD post-hoc test; ***, *P* < 0.001; ns, not significant.

Transcription of the key biosynthetic MGCs was previously found to be driven by multiple promoters (Supplementary Fig. S4, [20, 70]). Under conditions permitting magnetosome formation (= presence of iron and suboxic conditions), the *mamGFDCop* and *feoAB1op* are transcribed as single units, whereas multiple TSSs are present in *mms6op, mamXYop*, and *mamABop*, the latter comprising 17 genes and encoding all essential factors for magnetosome biosynthesis [20, 70]. As reported by Pang *et al.* (2024), in *nsrR_Mg_::GmR* transcription levels of magnetosome genes determined by qRT-PCR were decreased 0.3- to 23.8-fold for *mamG, mamH, mamI, mamY* and *feoA1* throughout growth, while transcription levels of *mms36, mms6*, and *feoB1* were reduced in *nsrR_Mg_::GmR* during early growth, but essentially reached those in the WT after 24 h growth. Activity of *lux*-reporter fusions with the major promoters *mamHp* and *mamIp* (referring to P*mamH* and P*(mamH)* in our study) of the essential *mamABop* from *M. gryph*. was also significantly increased in *E. coli* in the presence of *nsrR_Mg_* expressed from a plasmid. Based on these observations, Pang *et al.* (2024) suggested a role of NsrR*_Mg_* as direct transcriptional activator of magnetosome operons. This prompted us to investigate the putative regulatory effect of NsrR*_Mg_* on key magnetosome gene promoters directly in *M. gryph*. by our established *lux*-reporter system based on bacterial luminescence [70]. To this end, we probed fusions of the *luxAE* gene cassette to all magnetosome gene promoters tested by Pang *et al.* (2024) (Supplementary Fig. S4). All cassettes were insulated by two terminator sequences inserted immediately upstream of the promoters [70], and inserted into the chromosomes of WT and ∆*nsrR_Mg_* utilizing the *attTn7* site of *M. gryph*. by Tn7 transposition (Uebe *et al.*, manuscript in preparation). Strains harbouring respective reporter fusions exhibited similar transcriptional activity both in the WT as well as ∆*nsrR_Mg_* (Fig. 3 and Supplementary Table S6). The only exception was P*mamG*, which showed 6-fold increased activity in ∆*nsrR_Mg_* compared to WT. Overall, the activity of all promoters was not decreased in ∆*nsrR_Mg_*, but in the same range as observed previously [70], which again is in contrast to the observations by Pang *et al.* (2024).

### *Magnetospirillum gryphiswaldense* does not produce significant amounts of NO_2_^−^ from NH4

**Figure 4.**
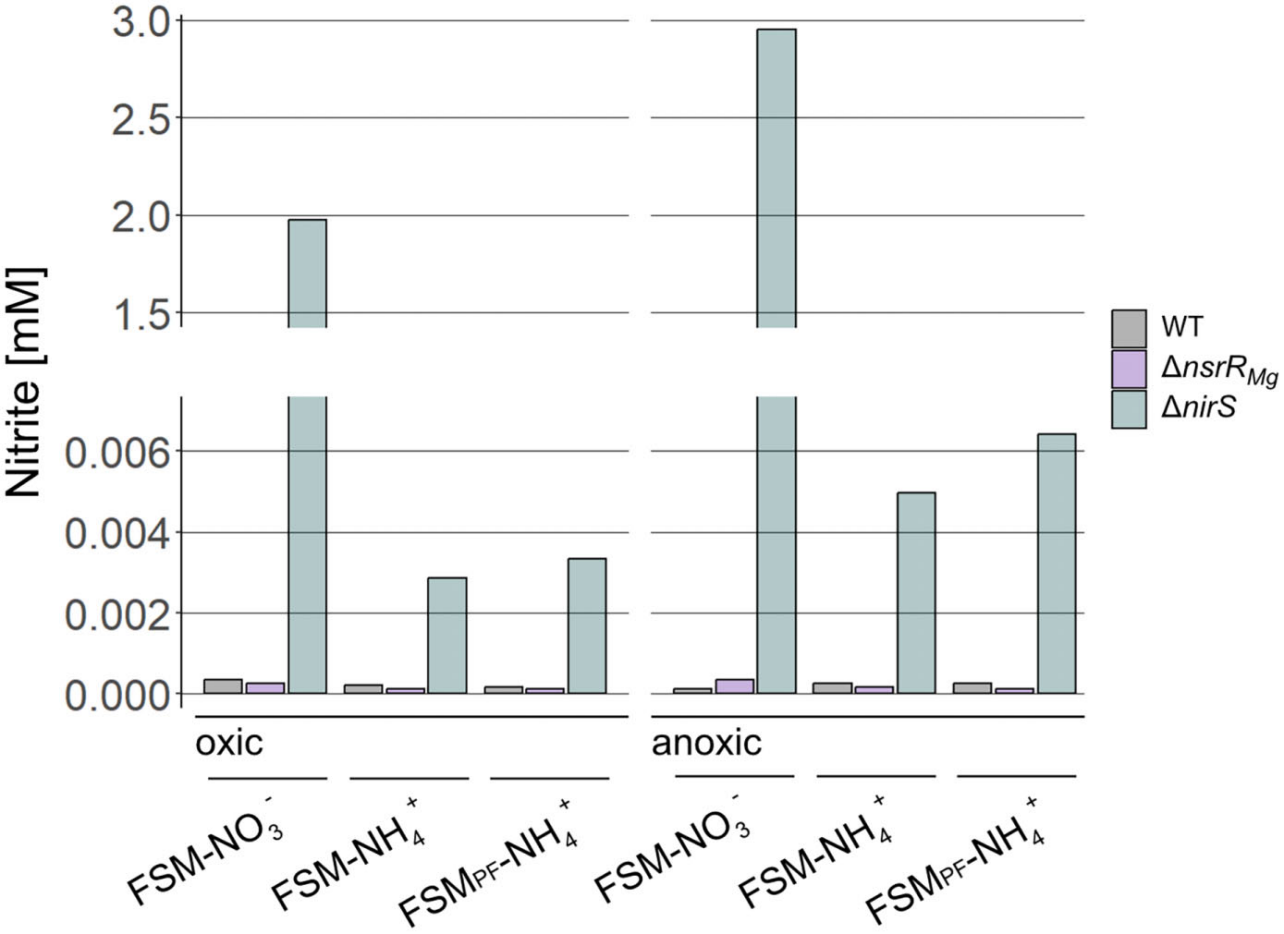
Nitrite content in WT (gray), Δ*nsrR_Mg_* (purple), and Δ*nirS* (green) after ca. 40 h growth in either FSM-NO_3_^−^, FSM-NH_4_^+_,_^or FSM_PF_-NH_4_^+^ under oxic (left) and anoxic (right) conditions.

To investigate the presence of a putative nitrification pathway, we tested for generation of the intermediate NO_2_^−^ in WT, Δ*nsrR_Mg_* and in strain Δ*nirS*, which accumulates nitrite upon production as it lacks the capacity to convert it further [27]. We tested all strains in FSM-NO_3_^−^, FSM-NH_4_^+^ and FSM_PF_-NH_4_^+^ under oxic as well as anoxic conditions. Under all conditions and in all media, WT and Δ*nsrR_Mg_* both did not accumulate significant amounts of NO_2_^−^ (between 0.1 and 0.4 µM NO_2_^−^) (Fig. 4). In contrast, similar to previous studies [27], Δ*nirS* accumulated 2 and 3 mM NO_2_^−^ in FSM-NO_3_^−^ under oxic and anoxic conditions, respectively (Fig. 4). However, in FSM-NH_4_^+^ and FSM_PF_-NH_4_^+^, Δ*nirS* accumulated only 2.9 and 3.3 µM NO_2_^−^ under aerobic and 5 and 6.4 µM NO_2_^−^ under anaerobic conditions, respectively, which is nearly 1000-fold lower than in FSM-NO_3_^−^. Since nitrification is an aerobic process [71, 72], and only very small amounts of NO_2_^−^ were detected in Δ*nirS* in FSM-NH_4_^+^ under both oxic as well as anoxic conditions, the amounts of detected intermediate nitrite are too low to account for significant nitrification activity.

### Bioinformatic analysis does not support the presence of a nitrification pathway in *Magnetospirillum gryphiswaldense*

Conspicuously, the presumable nitrification pathway suggested by Pang el al. (2024) also had remained previously undetected, e.g. by the KEGG pathway database. Apparently, its genomic prediction by Pang *et al.* (2024) mostly relied only on automated gene annotations, which however are prone to misprediction of gene functions. Indeed, as explained below, more sophisticated sequence and comparative domain analyses do not support the presence of canonical nitrification enzymes or a functional nitrification pathway in *M. gryph*. For example, MSR1_30780, hypothesized by Pang *et al.* to encode the ammonia monooxygenase subunit AmoA, belongs to the uncharacterized AbrB family (AbrB duplication, IPR017516, HHPred E-value 8.9e^−57^), which however is unrelated to ammonia monooxygenases (Supplementary Fig. S5A). Instead, the genomic context of MSR1_30780 indicates an operon together with genes unrelated to ammonium oxidation (Supplementary Fig. S5B). In addition, and importantly, *M. gryph*. lacks genes encoding putative AmoB and AmoC subunits, which are highly conserved across ammonium oxidizing bacteria and which are required to constitute a functional Amo enzyme [71–74]. *dnfABCD* genes, which have been implicated in heterotrophic nitrification in *Alcaligenes ammonioxydans* and *A. faecalis*, are also absent from the genome of *M. gryph*. [75]. Similarly, BLAST analyses using the presumptive hydroxylamine oxidoreductase homolog MSR1_25550 as a query revealed top hits to tetrathionate reductases, and consistently, MSR1_25550 clusters with the octaheme tetrathionate reductase (OTR) of *Shewanella oneidensis* [76] in protein cluster analysis, rather than with known HaoA proteins (Supplementary Fig. S5C). Finally, the putative NxrAB (nitrite oxidoreductase) proteins MSR1_20450/20440 are annotated as anaerobic dimethyl sulfoxide or tetrathionate reductase subunits and in BLAST analyses retrieved top hits to formate and sulfite dehydrogenases rather than to nitrite reductases. Also, both proteins cluster with sulfite dehydrogenase subunits SoeA and SoeB instead of canonical NxrA or NxrB, respectively (Supplementary Fig. S5D and E). Although according to Pang *et al.* (2024) being activated by NsrR*_Mg_*, none of the *M. gryph*. genes mentioned above are differentially transcribed between anoxic, microoxic, and fully oxic conditions, nor in response to the applied nitrogen source [20]. Taken together, these findings strongly suggest that each of the proposed nitrification pathway components is more likely involved in unrelated cellular processes, rather than in a nitrification pathway. In addition, whereas homologs of the NsrR regulator are present in all MTB, the capacity for nitrification does not appear to be conserved, even among close relatives, rendering its functional relevance unlikely.

## Discussion

We reassessed the role of the *nsrR_Mg_* gene encoding a putative NO-responsive transcriptional regulator on magnetosome biosynthesis in *M. gryph*. We found that under any tested conditions, NsrR*_Mg_* was not required for magnetosome biosynthesis, as cells continued to produce magnetosomes in WT-like amounts and sizes. This is in stark contrast to Pang *et al.* (2024) who reported that upon deletion of *nsrR*_*Mg*_, magnetosome formation was completely abolished under all tested conditions (i.e. with either ammonium or nitrate as nitrogen source). Moreover, we also failed to reproduce any regulatory effect of NsrR*_Mg_* on key magnetosome biosynthetic genes as reported by Pang *et al.* (2024). Since the original mutant strain *nsrR_Mg_::GmR* was no longer available from the Tian and Wen group upon our request, we were unable to experimentally reconcile the observed discrepancies. However, in the following, we discuss possible reasons for the conflicting results.

One possible explanation for the discrepancies could be that the two strains analyzed by Pang *et al.* (2024) and our study, although being descendants of the same *M. gryph*. type strain DSMZ 6361 [4, 5], might be genetically divergent, for instance by spontaneous rearrangements and mutations that may have occurred during serial lab cultivation. In fact, sequence comparison of the genome assembly (GenBank: NC_023065.1 [67]) referred to by Pang *et al.* (2024), and CP027526.1 [46] seems to indicate numerous mismatches, including the unlikely number of >69,000 putative single nucleotide polymorphisms (SNPs), >4,800 small indels as well as over > 800 larger structural differences like relocations, inversions, and insertions (accounting for ∼460 – 670 kb) (Supplementary Fig. S6). However, the earlier version of the genome assembly (NC_023065.1) with a size of 4,365,796 bp and 4,261 predicted genes was generated by using only Illumina Solexa and Roche 454 sequencing [67], which likely led to numerous misassemblies. This was later shown by re-sequencing of strain DSMZ 6361 by a combination of Illumina and PacBio single-molecule sequencing [46], the latter used because of its long read lengths (typically 15 – 25 kb) being more powerful for assembly of genomes with a high content of highly repetitive segments [77] such as present in *M. gryph*. In fact, this approach yielded a high-quality contiguous genome sequence with a somewhat smaller size (4,155,740 bp) and fewer (3,980) predicted genes (CP027526.1 [46]). In line with that, parallel genome sequencing of strain R3/S1, a spontaneous rifampicin resistant point mutant of strain DSMZ 6361 that had been repeatedly subcultivated in our lab for ca. 15 years (CP027527.1), revealed only 11 SNPs compared to CP027526.1 in the same study [46]. Thus, WT parent strains used by Pang *et al.* (2024) and in our study can be assumed to be genetically essentially identical, and genomic divergence is unlikely to account for the observed discrepancies and different phenotypes.

An obvious difference to Pang *et al.* (2024), however, is the genotype of our markerless *in frame* Δ*nsrR_Mg_* mutant, compared to *nsrR_Mg_::GmR* constructed by Pang *et al.* (2024) in which *nsrR_Mg_* was replaced by a gentamycin resistance cassette [36]. The insertion of a foreign DNA fragment may have led to the unintended introduction of internal promoters or transcriptional termination signals that may interfere with the expression of neighbouring genes, thus causing polarity effects [78]. However, as there is no indication for an operonic structure of *nsrR_Mg_*, this also seems to be not a very likely explanation for the observed differences.

A more likely reason for the loss of magnetosome formation in *nsrR_Mg_::GmR* of Pang *et al.* (2024) might be spontaneous second-site mutations in magnetosome genes co-occurring with the targeted deletion of *nsrR_Mg_*. For example, from the ∼120 transposable elements predicted in *M. gryph*., nearly 40 are encoded within the MAI and its adjacent regions, and several of them were found to be highly active [79], frequently causing spontaneous loss or inactivation of magnetosome biosynthetic genes in particular upon exposure to stress or prolonged storage [80–82]. Thus, given the high prevalence of such spontaneous mutations one could speculate that an overlooked second-site mutation had coincidently occurred in *nsrR::GmR*, which (instead of the deletion of *nsrR*) had caused the nonmagnetic phenotype. However, in this case, the mutation would be expected to be not complementable by reintroduction of *nsrR* on a medium copy plasmid in CnsrR_Mg_, as described by Pang *et al.* (2024). Thus, in the absence of the original mutant strains, this could not be clarified further, e.g. by genome resequencing of *nsrR::GmR* as well as the complemented strain CnsrR_Mg_.

As emphasized by Pang *et al.* (2024), denitrification is the only known pathway in *M. gryph*. to generate the regulatory NO signal sensed by NsrR*_Mg_*. Starting from NO_3_^−^ and catalyzed by the periplasmic nitrate reductase Nap [26], it generates nitrite (NO_2_^−^), which is then further reduced to NO by the Fe(II):nitrite oxidoreductase NirS [27, 83]. We found denitrification to be active in ∆*nsrR_Mg_*, as indicated by the production of gas (N_2_) in the presence of nitrate in our semiquantitative denitrification assay [27] (Supplementary Fig. S7). However, deletion mutants of either *nap* or *nirS*, in which NO formation is disrupted, are known to continue biomineralizing magnetite particles, if also with somewhat smaller sizes and more irregular shapes [26, 27]. Together with our bioluminescence reporter assays for magnetosome gene transcription that showed no significant difference between the WT and ∆*nsrR_Mg_*, this argues against the suggested role of NO as an essential signaling molecule, which would be required to ‘activate’ transcription of MGC genes.

According to Pang *et al.* (2024), *nsrR_Mg_::GmR* was unable to form magnetosomes when grown in either nitrate- or ammonium-containing mSLM medium, whereas the WT produced magnetosomes under both conditions. Notably, Pang *et al.* (2024) also observed slightly smaller crystals and fewer magnetosomes per cell in the WT in the presence of nitrate. In contrast, we detected WT-like magnetosome numbers in Δ*nsrR_Mg_* grown in FSM-NO₃⁻, whereas in FSM-NH_4_^+^ magnetosome numbers were reduced in both strains, with an even stronger effect in Δ*nsrR_Mg_*, consistent with earlier observations [18]. Notably, these differences became more pronounced when cells were cultivated in peptone-free medium. Besides reduced growth, magnetite biomineralization was strongly reduced when ammonium instead of nitrate was supplied as the sole source of nitrogen. Although elucidating the precise role of NsrR_Mg_ in *M. gryph*. was beyond the scope of this study, our findings suggest that the protein is not essential for denitrification nor biomineralization and that the observed lack of magnetosomes in *nsrR_Mg_::GmR* was rather an effect of suboptimal medium composition than genetic regulation. Instead, NsrR_Mg_ may contribute to the fine-tuned regulation of the denitrification pathway under some conditions and thereby facilitate NO detoxification as in other denitrifiers [43].

Finally, in the absence of exogeneous NO_3_^−^ in the mSLM medium used by Pang *et al.* (2024), a nitrification pathway was proposed in *M. gryph*. as a source for the endogenous NO_3_^−^, from which then the signal molecule NO is supposedly produced. If nitrate would indeed be produced via nitrification, one would expect its further reduction to N_2_ gas also in the absence of nitrate, but presence of ammonium in the medium. However, similar to Pang et al. (2024), we failed to detect gas production by the WT in FSM-NH_4_^+^. In addition, the detection of very small amounts of ^15^N-labeled NO_2_^−^ and NO_3_^−^ by Pang *et al.* (2024) was interpreted as an indication for active nitrification. However, no further intermediates were detected when ^15^N-NH_4_Cl was supplied as the sole nitrogen source, and the identification of ^15^N-labelled N_2_ in an experiment with mixed nitrogen sources (^15^N-NH_4_^+^ and ^15^N-NO_3_^−^) must be considered insufficient evidence for the presence of nitrification activity, but merely confirms the well-known fact that N_2_ is the final product of denitrification in the presence of NO_3_^−^. Furthermore, inhibition of nitrification by 3, 4-dimethylpyrazole-phosphate (DMPP) was observed only at extremely high concentrations (500 – 1500 µM) by Pang *et al.*, in contrast to its effect in known nitrifiers such as *N. europaea* and *N. multiformis*, where nitrification becomes already inhibited at concentrations as low as 1–10 µM [84]. Therefore, the observed impairment of magnetosome formation reported by Pang *et al.* (2024) may be due to nonspecific effects of artificially high DMPP concentrations on metabolism and biomineralization, potentially through its proposed metal-chelating activity [85]. Altogether, the data reported by Pang *et al.* seem to be insufficient to support the presence of a nitrification pathway. This is consistent with the results of our experiments, in which we were unable to detect significant production of NO_2_^−^ from NH_4_ (i.e. the first step in nitrification), and our bioinformatic analysis, which failed to confidently identify any genes required for nitrification in the genome of *M. gryphiswaldense*.

In summary, the results of Pang *et al.* (2024) and those presented in our study lead us to the following main conclusions: (i) NsrR is neither required for, nor the key regulator of magnetosome formation in *M. gryphiswaldense*. (ii) There is no compelling evidence for the existence of a functional nitrification pathway in *M. gryphiswaldense*.

## Supplementary Material

gkaf1422_Supplemental_File

## Data Availability

The data underlying this article are available in the article, in its online supplementary material and upon request.
