# Supplementary material for: No evidence for the putative nitric oxide sensor NsrR as a key regulator of magnetosome formation in Magnetospirillum gryphiswaldense
Source: Nucleic Acids Res. 2026 Jan 6;54(1):gkaf1422. doi: 10.1093/nar/gkaf1422 (PMC12774639; doi:10.1093/nar/gkaf1422)
Supplement: gkaf1422_Supplemental_File [file gkaf1422_supplemental_file.pdf]

SUPPLEMENTAL FIGURES

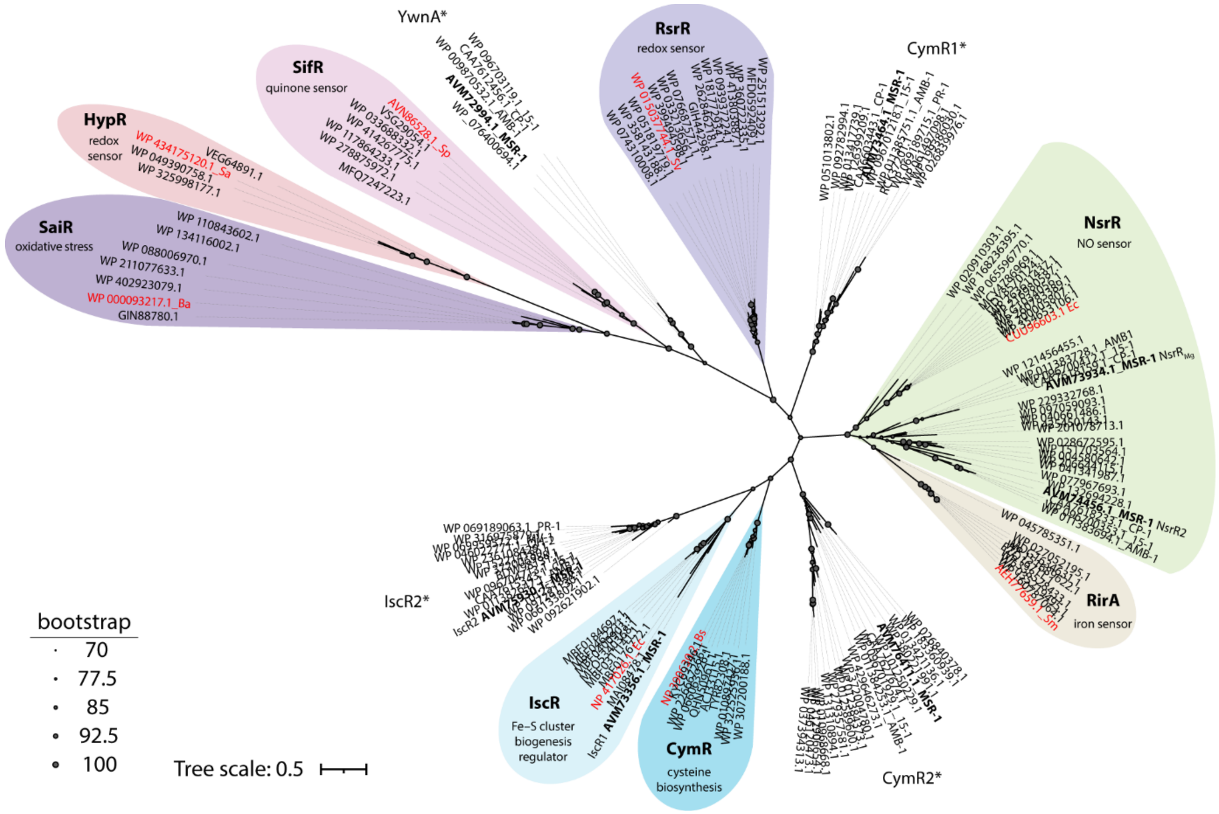

Fig. S1: Unrooted phylogenetic tree of the Rrf2 family inferred using the best-fitting LG+I+G4 substitution model. Characterized subfamilies are highlighted with colored backgrounds, and their known functions along with representative proteins shown in red font. Subfamilies marked with an asterisk are uncharacterized and named after the annotation of *M.gryph.* homologs within the respective group. Proteins encoded by *M.gryph.* are indicated in bold. Bootstrap support values (black dots) are denoted in the legend. Scale bar represents expected substitutions per site.

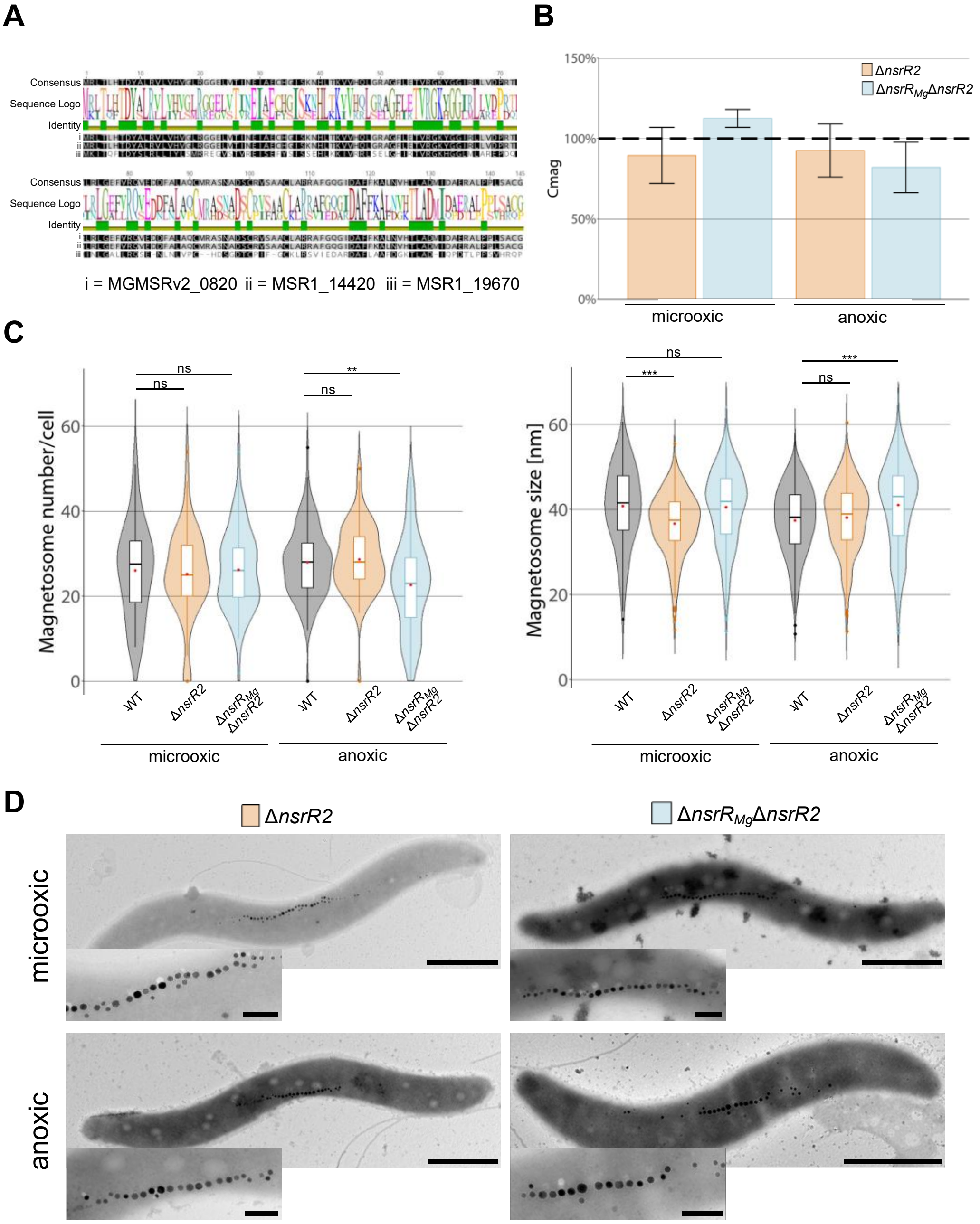

Fig. S2: Phenotypic analysis of Δ*nsr2* and Δ*nsrR*$_{Mg}$Δ*nsr2* compared to WT. (A) Protein alignment of MGMSRv2_0820, MSR1_14420 and MSR1_19670 (NsrR2). Similarity of sequence is highlighted with black (100%), darkgrey (80 to 100%), lightgrey (60 to 80%) and white (<60%) background. (B) Magnetic response of Δ*nsrR2* (orange) and Δ*nsrR*$_{Mg}$Δ*nsr2* (blue) compared to WT (dashed line) in FSM-NO_3^- under microoxic (left) and anoxic (right) conditions. Data represented for three biological replicates. (C) Violin plots displaying magnetosome number per cell (left) (n = 26 - 115) and magnetosome size (right, n 141 – 585) of WT (grey), Δ*nsrR2* (orange) and Δ*nsrR*$_{Mg}$Δ*nsr2* (blue) in FSM-NO_3^- under microoxic and anoxic conditions. Significance values were calculated by TukeyHSD post-hoc test; **, P value of less than 0.01; ***, P value of less than 0.001; ns, not significant. Boxplots within violin plots display the minimum, maximum, and median of each data set. Red points indicate mean. (D) Whole cell TEM images of Δ*nsrR2* (left) and Δ*nsrR*$_{Mg}$Δ*nsr2* (right) in FSM-NO_3^- under microoxic (top) and anoxic (bottom) conditions. Scale bars: 1 µm (whole cell), 200 nm (close-up).

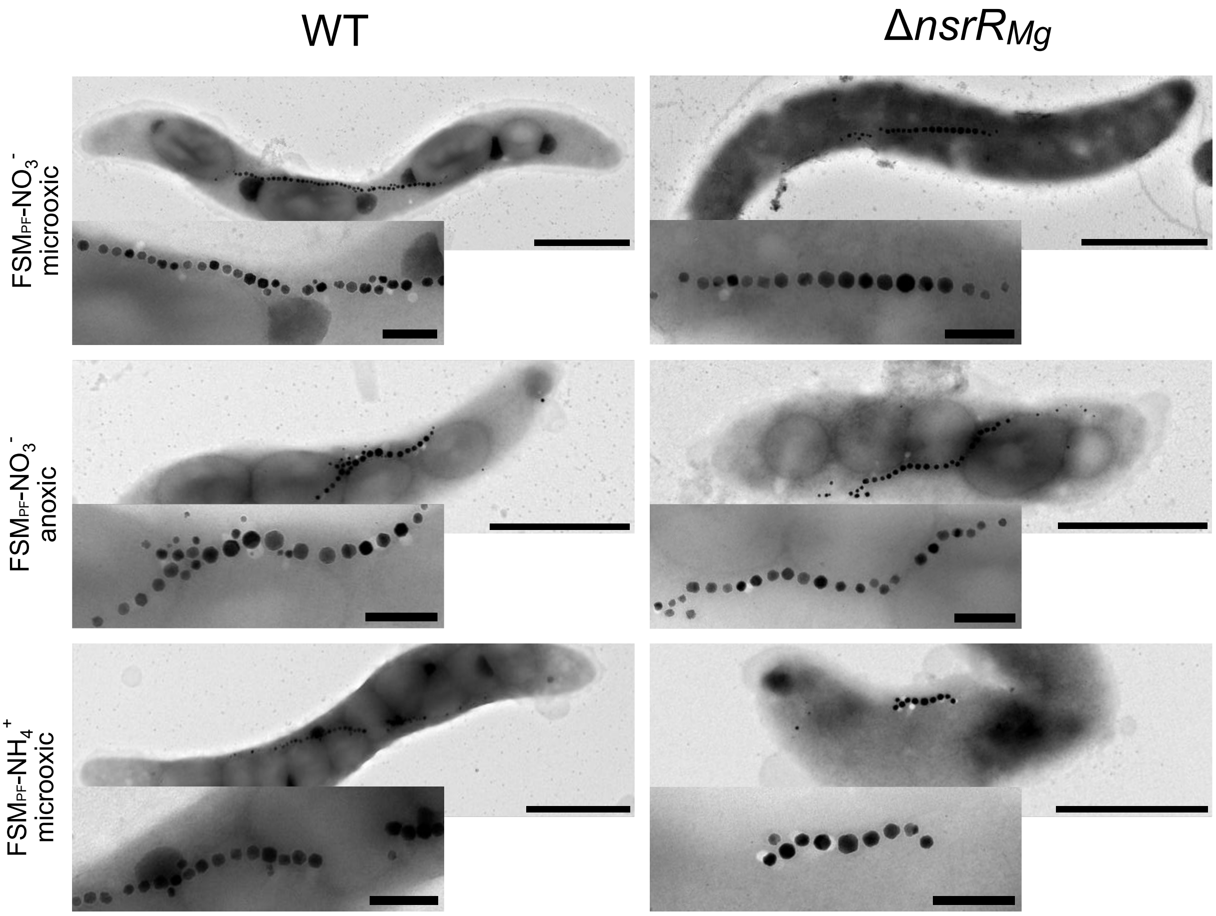

Fig. S3: TEM images of WT (left) and Δ*nsrR*$_{Mg}$ (right) cultivated in peptone-free FSM with either NO_3^- under microoxic (top) or anoxic (middle) conditions, or with NH_4^+ under microoxic conditions (bottom). Scale bars: 1 µm (whole cell), 200 nm (close-up).

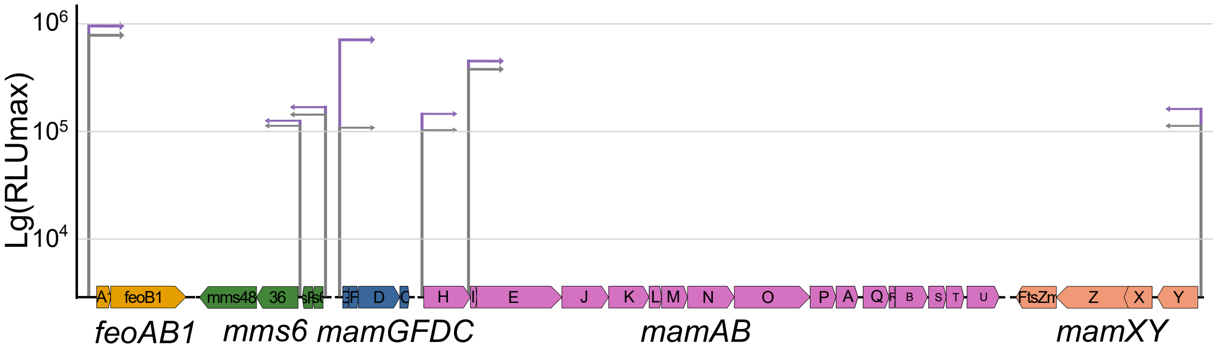

Fig. S4: Molecular organization and transcriptional architecture of the *M.gryph.* MGCs and position of the promoters, whose activities were confirmed by the bioluminescence assay, are indicated by grey (WT) and violet arrows ($\Delta nsrR_{Mg}$). Arrow height indicates mean promoter strength measured via bioluminescence assays in this study (see Fig. 3 and text for details).

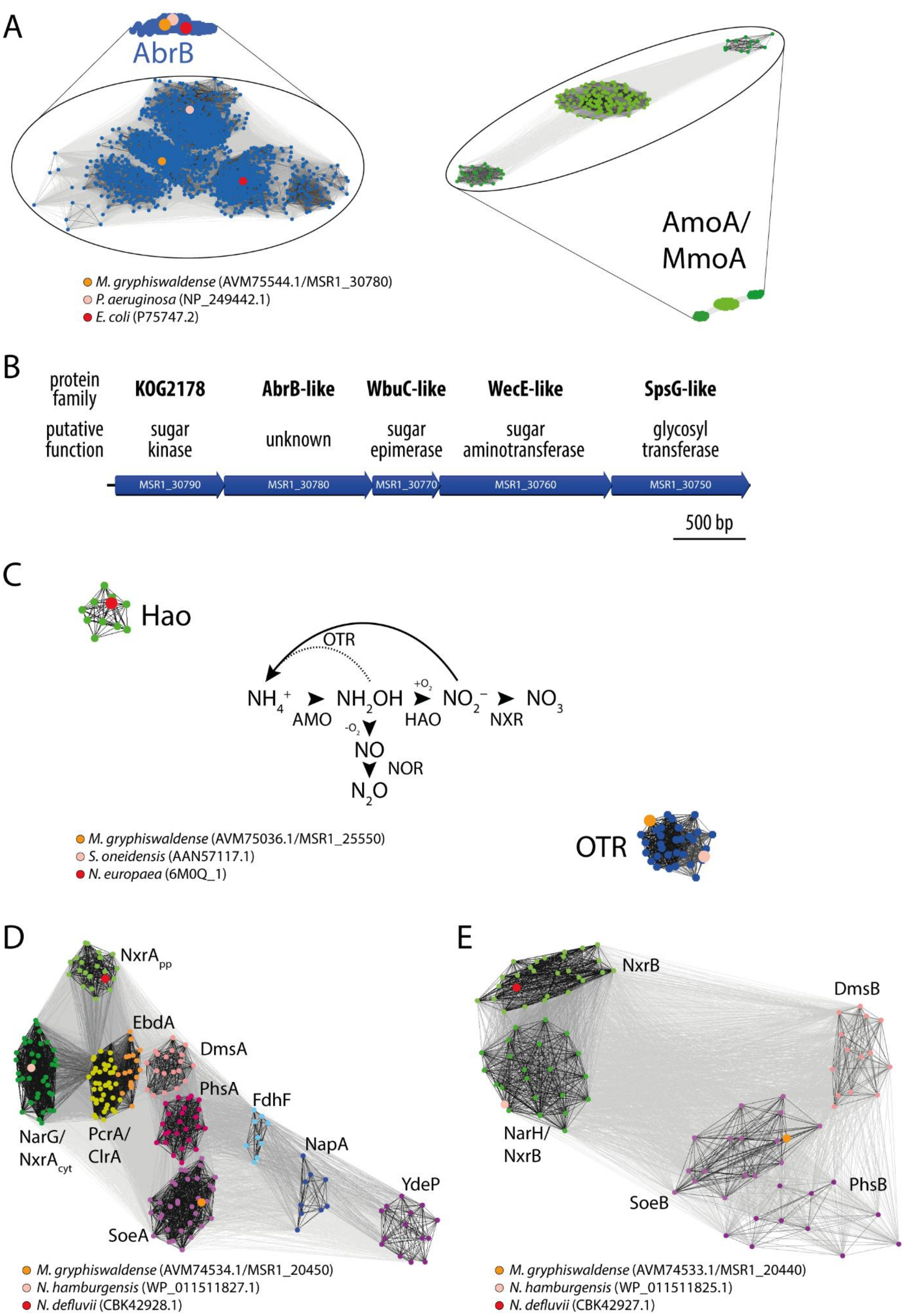

Fig. S5: Analysis of putative key enzymes of the nitrification pathway in *M. gryph*.
(A) Clustering analysis (CLANS) of 2,720 AbrB and AmoA family proteins. Protein sequences (colored dots) are positioned based on pairwise similarity. Sequences with higher

similarity are clustered more closely and connected by lines when BLASTp E-values are below 10^{-5}. Encircled regions show magnified views of individual clusters. Positions of representative AbrB family proteins from *M. gryph.*, *E. coli*, and *P. aeruginosa* are highlighted. Misleadingly, nearly one-third of all AbrB-family proteins in the Interpro database (IPR017516) are annotated as "(putative) ammonia oxidase", merely based on spurious local sequence similarities to the well-characterized *Nitrosomonas europaea* AmoA (21% in the case of MSR1_30780), but despite lacking experimental evidence for such activity. Notably, *Escherichia coli* and *Pseudomonas aeruginosa* also encode AbrB-family proteins yet are not known to oxidize ammonia. (B) Genomic context and molecular organization of the gene encoding the AbrB family protein MSR1_30780. Predicted protein families and their putative functions (indicated above the arrows) point to roles in sugar modification, suggesting involvement in lipopolysaccharide or extracellular polysaccharide biosynthesis. (C) Clustering analysis (CLANS) of 44 HaoA and OTR family proteins. Positioning and connections between proteins are shown as in panel A. Positions of representative proteins from *M. gryph.*, *S. oneidensis*, and *N. europaea* are highlighted. Notably, besides tetrathionate reduction, OTR from *S. oneidensis* has been shown to reduce nitrite to ammonia, thus catalyzing the reverse reaction of HaoA. (D) Clustering analysis (CLANS) of 216 molybdopterin-binding oxidoreductase family proteins. Positioning and connections between proteins are shown as in panel A. Positions of representative proteins from *M. gryph.*, *Nitrobacter hamburgensis*, and *Nitrospira defluvii* are highlighted. (E) Clustering analysis (CLANS) of 96 iron–sulfur cluster-binding β-subunits of molybdopterin-containing oxidoreductase family proteins. Positioning and connections between proteins are shown as in panel A. Positions of representative proteins from *M. gryph.*, *Nitrobacter hamburgensis*, and *Nitrospira defluvii* are highlighted.

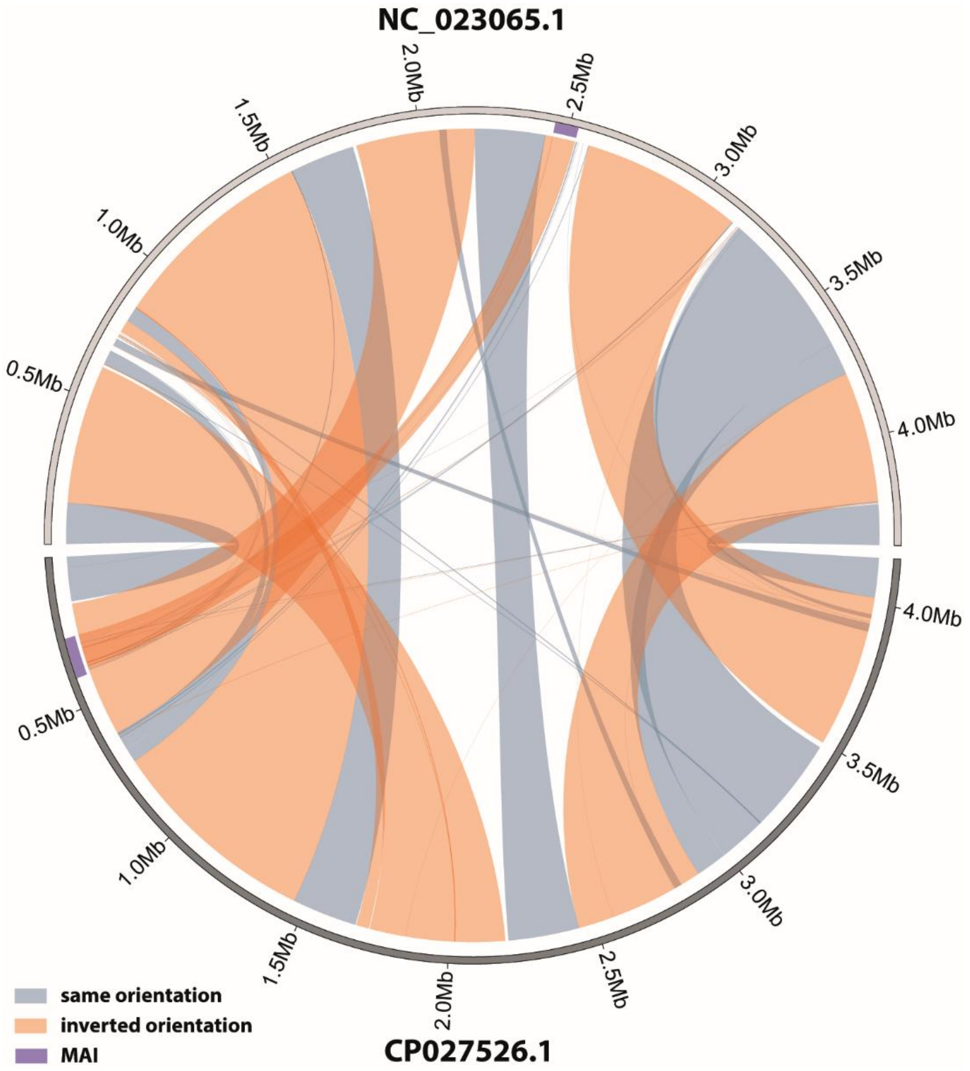

Fig. S6: Circos plot depicting presumable structural differences between *M.gryph.* genome sequences NC_023065.1 (4.37 Mb, Wang et al. 2014) and CP027526.1 (4.16 Mb, Uebe et al. 2018), which were both derived from the same type strain DSMZ 6361 (Ref Schleifer et al.1991). Links between the two circular chromosomes represent locally collinear blocks identified by Mauve whole genome alignments. Grey-blue ribbons indicate colinear (same orientation) regions, while orange ribbons mark regions with an inverted orientation. The width of each ribbon reflects the length of the aligned segment. Minor rearrangements and repetitive sequences such as transposases appear as thinner lines. As the two genome assemblies also differ by ~69,000 SNPs, they share an average nucleotide identity (ANI) of only 97.8%, which, for example, is markedly lower than the 99% ANI observed between the non-identical *E. coli* strains K-12 substr. MG1655 (U00096.3) and BL21 (CP010816.1).

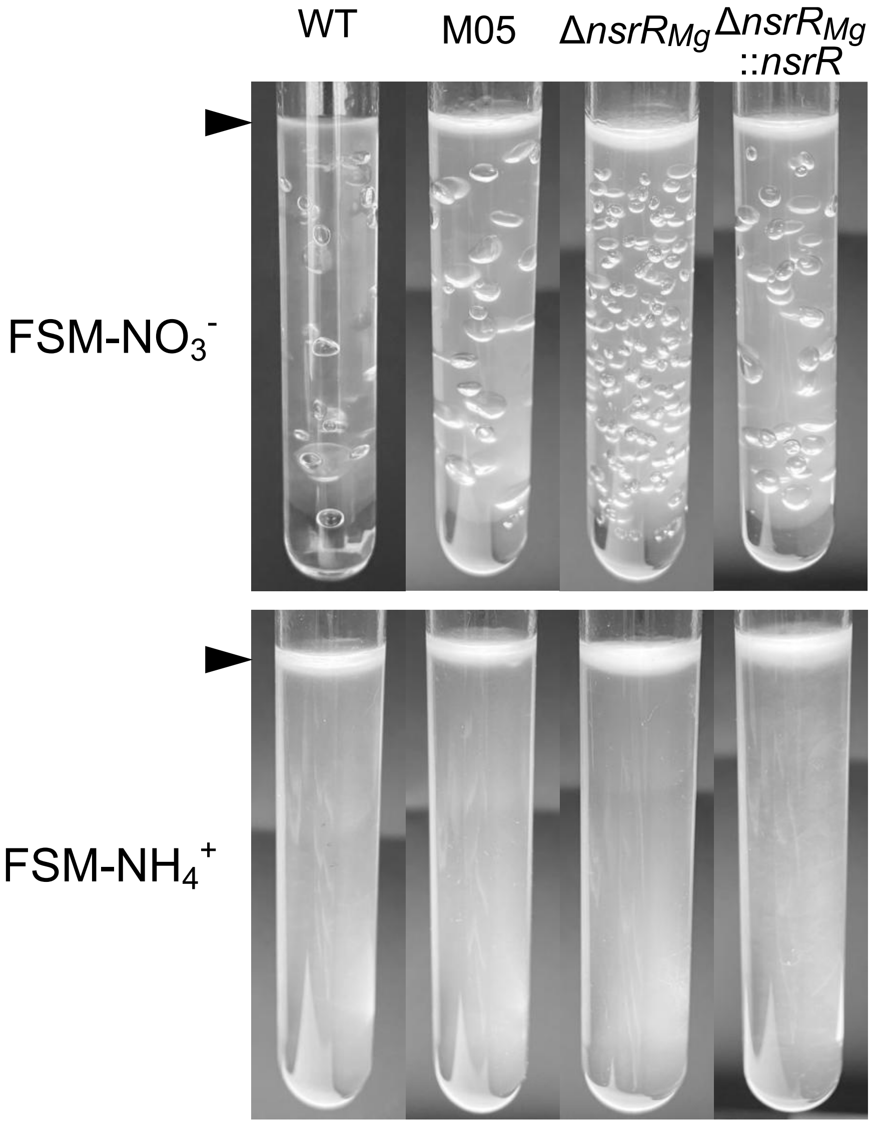

Fig. S7: Semi-quantitative denitrification assay in soft agar tubes. Strains were inoculated in FSM-NO_3^- (top) or FSM-NH_4^+ (bottom) and incubated under microoxic conditions. Arrows indicate a distinct band of aerobically growing cells just beneath the surface, while dispersed anaerobic growth occurs below along the entire depth of the agar tubes. N_2 gas formation can be seen as bubbles, which are not produced in the absence of NO_3^+.

SUPPLEMENTAL TABLES

Table S1. Bacterial strains and plasmids used in this work.

Strain or plasmid	Description	Source or reference
**Strains**		
Magnetospirillum gryphiswaldense	type strain, wildtype (WT)	DSMZ 6361 (Schüler/Köhler 1992, Schleifer 1991)
M. gryphiswaldense **M05**	non-magnetic mutant, *mms6op*, *mamGFDCop*, *mamABop* and *mamXYop*, MSR1_02690 - MSR11_02700, MSR1_03150 - MSR1_03880	Zwiener 2021
M. gryphiswaldense **Δ*nsrR*$_{Mg}$**	*nsrR*$_{Mg}$ deletion mutant	This study
M. gryphiswaldense **Δ*nsrR*$_{Mg}$::pBBR-*nsrR1***	Complemented *nsrR*$_{Mg}$ deletion mutant	This study
M. gryphiswaldense **Δ*nsrR2***	*nsrR2* deletion mutant	This study
M. gryphiswaldense **Δ*nsrR*$_{Mg}$Δ*nsrR2***	*nsrR*$_{Mg}$-*nsrR2* double deletion mutant	This study
E. coli **DH5α**	*F-supE44 ΔlacU169 (Φ80lacZDM15) hsdR17 recA1 endA1 gyrA96 thi-1 relA1*	Invitrogen
E. coli **WM3064**	*thrB1004 pro thi rpsL hsdS lacZΔM15 RP4-1360Δ(araBAD) 567 dapΔA1341::[erm pir]*. Donor strain for transformation by conjugation, α,ε -diaminopimelic acid (DAP) auxotroph.	William Metcalf, UIUC, unpublished
**Plasmids**		
**pOR-15a-*galK***	*npt galK tetR mobRK2*. General backbone vector for GalK counterselection.	Uebe, manuscript in preparation
**pOR-15a-*galK-nsrR1***	Deletion vector for *nsrR1.*	This study
**pOR-15a-*galK-nsrR2***	Deletion vector for *nsrR2.*	This study
**pBBR-MCS2**	Broad-host range *lacZ* promoter probe vector, *AmpR*, *KmR*; backbone for complementation vector.	Kovach et al. 1995
**pBBR-*nsrR1***	*nsrR* complementation vector.	This study
**pBamII-Tn7-P-*luxAE***	*KmR, AmpR, p15A ori, Tn7, tr2, T1, luxABCDE*; a plasmid for the transcriptional fusion of a promoter (P) and the lux operon. Suicide vector, a cassette is introduced by chromosomal insertion mediated by Tn7 into the attTn7 site.	Dziuba et al. 2021

Table S2. Primers used in this study.

Name/Number	Sequence (5' -> 3')	Description
**AW391**	GCCAGTGTCGAAATCCACAC	Amplification homologous downstream regions of *nsrR*$_{Mg}$
**AW394**	GCCATGGTTTCGATGCCTTC	Amplification homologous upstream regions of *nsrR*$_{Mg}$
**AW395**	ATACGACCAACGCCCTTAATGTAGTG TCGTCAGCCTG	Amplification homologous downstream regions of *nsrR*$_{Mg}$
**AW396**	GACACTACATTAAGGGCGTTGGTCGT ATTATCCT	Amplification homologous upstream regions of *nsrR*$_{Mg}$
**AW466**	GCCATGATCGATTGGTTGGC	Screening *nsrR*$_{Mg}$ deletion (flanking primer)
**AW467**	TGGAAGTGCAGGAACTCGAC	Screening *nsrR*$_{Mg}$ deletion (flanking primer)
**AW468**	TGGTGCATCAATTAGGGCGT	Screening *nsrR*$_{Mg}$ deletion (inside primer)
**AW469**	ACGCCCTAATTGATGCACCA	Screening *nsrR*$_{Mg}$ deletion (inside primer)
**AW471**	GGGGAATTCATGCGACTGACGCTTCA CAC	Amplification *nsrR*$_{Mg}$ for complementation
**AW472**	AGGAAGCTTTCAGCCGCAAGCTGAC AGG	Amplifikation *nsrR*$_{Mg}$ for complementation
**AW483**	TGGCCGAACGTCTGCCCTA	Amplification homologous downstream regions of *nsrR2*
**AW484**	GACTTTGGCTGATCTCAGTCCACCGG CAGCCATA	Amplification homologous downstream regions of *nsrR2*
**AW485**	TGCCGGTGGACTGAGATCAGCCAAA GTCAGGTTTC	Amplification homologous upstream regions of *nsrR2*
**AW486**	ACGAATCGGCCATCTTGCA	Amplification homologous upstream regions of *nsrR2*
**AW487**	CATGTCCCGAGGCAAAGAGT	Screening *nsrR2* deletion (flanking primer)
**AW488**	CATCACCTCGACCGATCTGG	Screening *nsrR2* deletion (flanking primer)
**AW489**	GAACTGGGGCATATCCGCAC	Screening *nsrR2* deletion (inside primer)
**RU177**	ATCGGTGACGGTGGAATAGTCCGC	Screening POI-*luxAE* genome insertion
**RU178**	GGCATGGATGATGCTGTCCCTGGT	Screening POI-*luxAE* genome insertion
**RU841**	TTTCTACGTGTTCCGCTTCC	Screening and sequencing pOR-15a-*galK*-*nsrR*$_{Mg}$
**RU1852**	CGCTATAATGACCCCGAAGCAGG	Screening and sequencing pOR-15a-*galK*-*nsrR*$_{Mg}$

Table S3. Final OD for all tested strains after growth under different conditions given as mean (± standard deviation (SD)) of 3 independent replicates. With * indicated conditions were only tested once.

OD565	Oxic*					Microoxic					Anoxic				
	FSM-NO_3^-	FSM-NH_4^+	mSLM	FSM_{PF}-NO_3^-	FSM_{PF}-NH_4^+	FSM-NO_3^-	FSM-NH_4^+	mSLM*	FSM_{PF}-NO_3^-	FSM_{PF}-NH_4^+	FSM-NO_3^-	FSM-NH_4^+	mSLM*	FSM_{PF}-NO_3^-	FSM_{PF}-NH_4^+
WT	0.66	0.79	0.03	ND	ND	0.26 ± 0.06	0.15 ± 0.05	0.04	0.32 ± 0.02	0.06 ± 0.01	0.18 ± 0.01	0.06 ± 0.01	ND	0.20 ± 0.12	ND
M05	0.30	0.26	ND	ND	ND	0.22 ± 0.02	0.13 ± 0.04	ND	0.10 ± 0.01	0.03 ± 0.01	0.15 ± 0.03	0.06 ± 0.01	ND	0.10 ± 0.01	ND
$nsrR_{Mg}$	0.63	0.70	0.03	ND	ND	0.24 ± 0.02	0.16 ± 0.07	0.03	0.19 ± 0.05	0.02 ± 0.01	0.15 ± 0.07	0.06 ± 0.02	ND	0.20 ± 0.1	ND
Δ*$nsrR_{Mg}$::$nsrR_{Mg}$*	0.64	0.71	ND	ND	ND	0.26 ± 0.04	0.15 ± 0.05	ND	ND	ND	0.22 ± 0.09	0.1 ± 0.04	ND	ND	ND
Δ*nsrR2*	ND	ND	ND	ND	ND	0.24 ± 0.02	ND	ND	0.23 ± 0.05	0.26 ± 0.01	0.14 ± 0	ND	ND	0.21 ± 0.04	ND
Δ*$nsrR_{Mg}$*Δ*nsrR2*	ND	ND	ND	ND	ND	0.19 ± 0.03	ND	ND	0.20 ± 0.02	0.14 ± 0.04	0.09 ± 0.01	ND	ND	0.16 ± 0	ND

Table S4. Final magnetic response (Cmag) for all tested strains after growth under different conditions given as mean (± SD) of 3 independent replicates. With * indicated conditions were only tested once.

Cmag	Oxic*					Microoxic					Anoxic				
	FSM-NO_3^-	FSM-NH_4^+	mSLM	FSM_{PF}-NO_3^-	FSM_{PF}-NH_4^+	FSM-NO_3^-	FSM-NH_4^+	mSLM*	FSM_{PF}-NO_3^-	FSM_{PF}-NH_4^+	FSM-NO_3^-	FSM-NH_4^+	mSLM*	FSM_{PF}-NO_3^-	FSM_{PF}-NH_4^+
WT	0.1	0.1	0.5	ND	ND	1.5 ± 0.1	1.0 ± 0.2	0.7	1.0 ± 0.1	0.7 ± 0	1.5 ± 0.2	1.3 ± 1.2	ND	1.1 ± 0	ND
M05	0	0	ND	ND	ND	0 ± 0	0 ± 0	ND	0 ± 0	0 ± 0	0 ± 0	0 ± 0	ND	0 ± 0	ND
$nsrR_{Mg}$	0	0.1	0.3	ND	ND	1.4 ± 0.2	1.1 ± 0.1	0.3	0.7 ± 0.2	0.3 ± 0.1	1.2 ± 0.2	1.0 ± 0.3	ND	0.8 ± 0.2	ND
Δ*$nsrR_{Mg}$::$nsrR_{Mg}$*	0	0.1	ND	ND	ND	1.0 ± 0.1	0.9 ± 0.0	ND	ND	ND	1.1 ± 0.2	0.6 ± 0.4	ND	ND	ND
Δ*nsrR2*	ND	ND	ND	ND	ND	1.4 ± 0.2	ND	ND	1.2 ± 0.2	0.8 ± 0.1	1.3 ± 0.2	ND	ND	1.3 ± 0.1	ND
Δ*$nsrR_{Mg}$*Δ*nsrR2*	ND	ND	ND	ND	ND	1.7 ± 0.1	ND	ND	0.8 ± 0.1	0.5 ± 0.3	1.2 ± 0.2	ND	ND	1.0 ± 0.2	ND

Table S5. Mean magnetosome number per cell and mean magnetosome size (± SD) for all tested strains and all conditions.

Strain	Condition	Medium	Mean mag/cell ± SD	Mean magsize ± SD
**WT**	microoxic	nitrate	26 ± 11 (n = 26)	41 ± 9 (n = 141)
***nsrR*$_{Mg}$**			25 ± 10 (n = 86)	37 ± 8 (n = 380)
**Δ*nsrR*$_{Mg}$::*nsrR*$_{Mg}$**			16 ± 12 (n = 84)	40 ± 11 (n = 339)
**Δ*nsrR2***			25 ± 11 (n = 62)	37 ± 7 (n = 346)
**Δ*nsrR*$_{Mg}$Δ*nsrR2***			26 ± 9 (n = 76)	38 ± 8 (n = 585)
**WT**		ammonium	14 ± 6 (n = 30)	36 ± 9 (n = 106)
**Δ*nsrR*$_{Mg}$**			9 ± 7 (n = 15)	35 ± 9 (n = 78)
**Δ*nsrR*$_{Mg}$::*nsrR*$_{Mg}$**			15 ± 7 (n = 21)	38 ± 9 (n = 99)
**WT**	anoxic	nitrate	28 ± 8 (n = 115)	37 ± 8 (n = 495)
**Δ*nsrR*$_{Mg}$**			28 ± 9 (n = 70)	38 ± 10 (n = 375)
**Δ*nsrR*$_{Mg}$::*nsrR*$_{Mg}$**			17 ± 12 (n = 84)	38 ± 10 (n = 382)
**Δ*nsrR2***			29 ± 8 (n = 61)	41 ± 9 (n = 356)
**Δ*nsrR*$_{Mg}$Δ*nsrR2***			23 ± 11 (n = 71)	41 ± 10 (n = 407)

Table S6: Characteristics of tested promoters. For each promoter, RLUmax values (mean ± SD) are shown for three representative clones.

**Promoter**	**Fragment sequence 5' -3'**	**Fragment length (bp)**	**Background strain**	**Activity, maximal emitted light (RLUmax)**		
				**Clone 1**	**Clone 2**	**Clone 3**
P*feoAB1*	GTGCCGAGGGAAAACATCTGCTTGCTCCCATCGCAAGTTGGCTGAATGA<br>CCTTTGTGATGCGACTTGCCGGGTGGCCCAACATTGGATTTTGCGCCTT<br>GACTCGGCAGCAAGATTGAGGGAAGATGCGCTGAATTAATCGCAACTCA<br>TTCGCAATTGCTTTCGATTATTGCGGCATTAACAAATAAGGAGGAGTTC<br>CAGG	200	WT	902333 ± 18716	682686 ± 63250	681656 ± 10870
			$\Delta nsrR_{Mg}$	1043498 ± 26731	934340 ± 23472	648105 ± 88469
P*mms36*	GGTTCGGCCTCGGGTGATTTACGTCTGATGCCCGGCTGTGCAGGGCATC<br>AGCTGTTTCAGGACCGTTTCATCTATAATATTAATGTCTTGCCAAAGGG<br>ATCGGTGATCGTGAAAATCGCCGTTGCGGGGCGGTTGGGCCCGGTTGCA<br>GGCCGCGCAGCTAGCGGAAGCGGAGCCA	175	WT	173961 ± 12950	129036 ± 1430	123359 ± 5281
			$\Delta nsrR_{Mg}$	156832 ± 9959	153937 ± 5229	132422 ± 6637
P*mms6*	ACCTGTCAATTCCTTCCCTGGTCATCGCTGCGCGCCATGCCTGCCAAAC<br>AGGGTGCTTTGCTCACGGTGGCAGATTTCTGTGGGGAGGTTTAAGGTGG<br>TGGTGGTCATTGGCGTTAAGGGATCGTCCCGTGCTTACTCTTTGGGATT<br>TGTCGACAATTATTGGGCTTGTGGTTTTGGCGGGAGCTGCGGTGCTTCG<br>CTGTGTCCACAAGAACCGCGACAGCGGGGCCGGGTGCAACAGGGGCTGC<br>TGTCAAAACCCGCGCAGAGGCAAATGCGAATAACCCGCAGCTAGCGGGG<br>GCGGTGCAGATAATAATCGCTCGTAACTTCCATGTAATAGTGTTTTTTA<br>GCCCCATACAGGTTGTCCTATTCGGCAACTCATGGTATTGAATTGTATG<br>TCTGGTGAGGCTGATGTTTTTCAGTCCCGCACTACTTGATTGCTAAGGA<br>GAACTCTG	450	WT	184949 ± 3824	137819 ± 5458	121568 ± 1463
			$\Delta nsrR_{Mg}$	222770 ± 4197	160049 ± 10721	126719 ± 5556
P*mamG*	CAACTTTTTCGCTTTACTAGCTCTTAGTTCTCCAATAAATTCCCTGCGT<br>CGATTTTAAGGGGCAGAGAGGGAATCGTGCATACA<br>CTTGCCGGAGATCAG	99	WT	102034 ± 5959	125183 ± 11061	121527 ± 1878
			$\Delta nsrR_{Mg}$	840199 ± 26608	728625 ± 11796	537133 ± 12607
P*mamH*	GGCATCCTGATCGGTAGGCGATGGCGCAAAGATGTGACGTCTTGTTTCG<br>AGCAGATGCGCGAAATGTGCTAGCGCGCTAAGTGTGCCACATTGCGAGG<br>TTCCTTCGTATGAACCCAGTTTTTATGGCTTGTCAACCGACCTCGATTC<br>GTTGCTATAGTCCGTGCGAATTGGAGGTGAATTGTGACGGGA	189	WT	120809 ± 10238	106435 ± 4681	91112 ± 3222
			$\Delta nsrR_{Mg}$	232203 ± 6966	117949 ± 10622	91174 ± 4940
P(*mamH)*	GTTCAGATTGGTGGGTTTTTATTTGATTACATTGGCCCTCCTGCGCCTT<br>TTATCTTTACGGGCGTTGGAAATTTGATTATATCAGCGTATGCGTTGCG<br>TCTTGTGAAAAGTGAGGCGCGTGGGGTTGGCGGGGGTAACACCCCAGGG<br>GATGACGAAGTGGCCTAGCGCCGCTTGTGTTCTGTATCAAGACTGGAGA<br>ACGTTT	202	WT	598886 ± 61441	462466 ± 26946	300995 ± 6868
			$\Delta nsrR_{Mg}$	719689 ± 25104	470414 ± 15082	360020 ± 6775
P*mamY*	GGCAGCCTCATTTAAACATTCAGGACGCGCTGCCATATTATACTATTCC<br>GAAATTAATACTTAGCACCACTTTCCCAACGGACTCTTAGGCAATGCAG<br>GTCCTCACAAACAAAAGGCGGTGTTGCGATTAATGCGCATCTTCCGGTA<br>TTGACGGACTCGCCACAAAGCCATATCTATCCCTTGTGTCCAAGCTACC<br>TTGCCGGTCAGCTTGCTGTTACTGTGGCAGTATCCTATACACACCAGAA<br>CATTTTGGCGACCACCACAGCCACCACGGGAGCAGCCCTT	285	WT	148550 ± 3130	139917 ± 5977	110572 ± 2018
			$\Delta nsrR_{Mg}$	226616 ± 5950	218000 ± 10262	145340 ± 33529
